# On the Shortfall of Tail-Based Entropy and Its Application to Capital Allocation

**DOI:** 10.3390/e27111153

**Published:** 2025-11-13

**Authors:** Pingyun Li, Chuancun Yin

**Affiliations:** School of Statistics and Data Science, Qufu Normal University, Qufu 273165, China; pingyun31@gmail.com

**Keywords:** tail-based entropy, expected shortfall, variability measure, capital allocation, tail risk

## Abstract

We introduce and study the shortfall of tail-based entropy (STE), a tail-sensitive risk functional that combines expected shortfall (ES) and tail-based entropy (TE). Beyond the tail mean, STE imposes a rank-dependent penalty on tail variability, thereby capturing both the magnitude and variability of tail risk under extremes. The framework encompasses several shortfall-type measures as special cases, such as Gini shortfall, extended Gini shortfall, shortfall of cumulative residual entropy, shortfall of right-tail deviation, and shortfall of cumulative residual Tsallis entropy. We provide equivalent characterizations of STE, derive sufficient conditions for coherence, and establish monotonicity with respect to tail-variability order. As an application, we investigate STE-based capital allocation, deriving closed-form allocation formulas under elliptical and extended skew-normal distributions, along with several illustrative special cases. Finally, an empirical analysis with insurance company data illustrates the implementation and evaluates the performance of the allocation rule.

## 1. Introduction

In modern risk management, a large number of risk measures have been proposed. These measures are mappings from random losses to real numbers. Among tail-based measures, value-at-risk (VaR) and expected shortfall (ES) are arguably the most widely used. We now recall the definitions adopted here. Let *X* be a loss variable with cumulative distribution function (cdf) $F_{X}$. For a confidence level p∈(0,1), the VaR at level *p* is defined as ${\mathrm{VaR}}_{p}\left(X\right)=F_{X}^{-1}\left(p\right)$, where $F_{X}^{-1}\left(p\right)=\inf\left\{x\in R:F_{X}\left(x\right)\geq p\right\}$ denotes the left-continuous inverse of $F_{X}$, with the convention inf⌀:=∞. The ES at level *p* is defined as

(1) $${\mathrm{ES}}_{p}\left(X\right)=\frac{1}{1-p}\int_{p}^{1}{\mathrm{VaR}}_{q}\left(X\right)\;dq.$$

For continuous random variables, ES coincides with the conditional tail expectation (CTE), which is given by

(2) $$CTE_{p}\left(X\right)=E\left[X|X>x_{p}\right],$$

where $x_{p}=VaR_{p}\left(X\right)$. From here to the end of the section, we assume $P(X>x_{p})>0$ so that (2) is properly defined.

Due to their nature, the above risk measures focus primarily on the expected risk level, negelecting the variability of tail risk in extreme market conditions. In practice, when pricing, capital, or stress decisions are sensitive to tail variability, it is natural to include an additional measure that captures this within-tail uncertainty. Recent work addresses this gap by incorporating entropy-based measures of variability into risk assessment. Rooted in information theory, these measures quantify uncertainty within the tail. For instance, Chakraborty and Pradhan [1] proposed and explored weighted cumulative Tsallis residual entropy and past entropy measures, along with their dynamic versions. Psarrakos et al. [2] developed a family of variability measures based on distorted weighted cumulative residual entropy through sensitivity analysis of distortion risk measures, establishing their theoretical properties, covariance representation. Rajesh and Sunoj [3] introduced an alternative form of the cumulative residual Tsallis entropy of order α and proved some results with applications in reliability. Calì et al. [4] studied alternatives to Shannon entropy by investigating cumulative residual and past entropies, establishing their generalized properties and applications in coherent systems. Mohammadi and Hashempour [5] introduced dynamic cumulative residual extropy inaccuracy as an extension of dynamic cumulative residual extropy, proposed a weighted variant, and studied its properties. Morever, Zardasht et al. [6] developed a goodness-of-fit test for exponentiality using the empirical cumulative residual entropy and established its asymptotic normality. For other significant contributions to entropy-based risk measures, see also Haberman et al. [7], Yin et al. [8], Hashempour et al. [9], and Toomaj et al. [10].

To capture both tail risk level and variability, a stream of work combines variability measures with ES or CTE in shortfall-type constructions. For example, Furman and Landsman [11] introduced the tail standard deviation (TSD) as a combination of CTE and standard deviation (SD). When CTE is replaced by ES, the TSD measure transforms into the standard deviation shortfall (SDS). Recently, there has been growing interest in integrating tail-based entropy measures with expected shortfall for a more comprehensive assessment of tail risk. Furman et al. [12] proposed Gini-type risk and variability measures and developed corresponding economic capital allocation rules. Hu and Chen [13] introduced a family of coherent variability measures and constructed a new risk measure shortfall of cumulative residual entropy (CRES). Berkhouch et al. [14] proposed the extended Gini shortfall (EGS), which maintains coherence and comonotonic additivity while effectively capturing tail risk by incorporating risk preference considerations. Ben Hssain et al. [15] further demonstrated the practical application of EGS in portfolio selection through a convex optimization framework, where the objective was to minimize portfolio risk subject to reward and budget constraints. Zuo and Yin [16] developed covariance and Choquet integral representations for some entropy measures and, based on this, introduced new risk measures, including a shortfall of cumulative residual Tsallis entropy (CRTES) and shortfall of right-tail deviation entropy (RTDS), offering new tools for understanding and quantifying tail risk. Furthermore, Zuo and Yin [17] discussed worst-case distortion riskmetrics for general distributions under partial information (mean and variance), and provided applications to shortfalls of tail-based entropy measures.

Once a portfolio-level tail risk measure has been specified for an aggregate loss composed of individual units, a standard follow-up is capital allocation based on that measure. Capital allocation is essential for risk pricing, budgeting, and performance evaluation in insurance business lines. Among existing principles, the Euler allocation principle stands as one of the most influential approaches. As shown by Tasche [18,19], it justifies the use of CTE for evaluating the performance of each risk unit, where performance is defined as the risk-adjusted return on capital. Kim and Kim [20] derive analytic formulas of the CTE allocation for the class of multivariate normal mean-variance mixture distributions. Li et al. [21] explore a generalization of ES-based capital allocation, which is a class of allocation rules based on Orlicz risk measure with different Young functions. Marri and Moutanabbir [22] explores risk aggregation and capital allocation with a mixed Bernstein copula, deriving closed-form expressions for TVaR and TVaR-based allocations. Several notable contributions have been made to alternative capital allocation principles. For instance, Dhaene et al. [23] introduced a unified approach based on a distance-minimizing principle for optimal capital allocation. Chen et al. [24] developed a dynamic framework incorporating reallocation costs, demonstrating its ability to maintain stability across periods. Boonen [25] proposed the τ-value for risk capital allocation and established its desirable properties. Yang et al. [26] introduced a generalized tail mean-variance model based on Bregman divergence, offering a new perspective on capital allocation. Additional research can be found in the works by Furman and Zitikis [27], Ostaszewski and Xu [28], Xu and Mao [29], Xu [30], Bauer and Zanjani [31], Balog et al. [32], Furman et al. [33], Boonen et al. [34], Chong et al. [35], Grechuk [36], Jiang et al. [37], Wei and Hu [38,39], Guo et al. [40], Mastrogiacomo and Rosazza Gianin [41], and Li and Yin [42].

Despite these advances, existing entropy-based shortfall measures face two key limitations. First, they lack a unified theoretical framework connecting different entropy functionals. Second, their capital allocation properties remain underexplored, limiting practical implementation. To address these limitations, this paper proposes shortfall of tail-based entropy (STE) risk measure, a tail-sensitive risk functional that combines expected shortfall (ES) and tail-based entropy (TE). Beyond the tail mean, STE imposes a rank-dependent penalty on tail variability, thereby capturing both the magnitude and variability of tail risk under extremes. The framework accommodates several shortfall-type measures as special cases, such as Gini shortfall (GS), EGS, CRES, RTDS, and CRTES. We develop the theoretical properties of STE and, as an application, propose the associated STE-based capital allocation rule. The main contributions of this paper include the following: First, we provide equivalent characterizations, derive sufficient conditions for coherence, and establish monotonicity with respect to a tail-variability order. Second, we characterize the STE-based capital allocation rule, analyze its fundamental properties, derive closed-form formulas under a centered-regression representation, and establish the connection with the distance-minimization framework of Dhaene et al. [23]. Third, we derive explicit expressions for STE allocation under elliptical and extended skew-normal distributions, and conduct empirical research based on insurance data, with results showing that this method outperforms traditional ES and SDS methods in identifying tail risk variability and capital allocation.

The remainder of this paper is organized as follows. Section 2 introduces the necessary preliminaries. Section 3 presents TE and its properties as the foundation for the STE, and analyzes the theoretical properties of STE. Section 4 develops the STE-based allocation and explores its theoretical properties. Section 5 derives the explicit expressions for the allocation rules under elliptical and extended skew-normal distributions, and investigates special cases of STE measures. An empirical analysis is provided in Section 6, and some concluding remarks are given in Section 7. Detailed proofs and auxiliary lemmas are provided in the Supplementary Materials).

## 2. Preliminaries

We work on an atomless probability space (Ω,F,P). For k∈[0,∞), let $L^{k}=L^{k}\left(\Omega ,F,P\right)$ denote the set of random variables with finite *k*th-moment (and let $L^{\infty }$ be the set of essentially bounded random variables). Throughout, positive (negative) values of $X\in L^{0}$ represent financial losses (profits). For a random variable *X*, let $F_{X}$ denote its cumulative distribution function (cdf), and let $U_{X}$ denote any uniform [0,1] rv such that the equation $F_{X}^{-1}\left(U_{X}\right)=X$ holds almost surely. The existence of $U_{X}$ is given in Lemma A.28 of Föllmer and Schied [43]. We write $X\overset{d}{=}Y$ to denote that *X* and *Y* are equal in distribution. Let $h^{\prime }$ denote the right derivative of *h*. Notation

$$H=\left\{h:[0,1]\to R\;|\;h\;\mathrm{is}\;\mathrm{concave}\;\mathrm{and}\;h(0)=h(1)=0\right\},$$

and $I_{A}\left(\cdot \right)$ is the indicator function of set *A*. Throughout the present paper, we restrict our attention to random variables in $L^{2}$.

In this section, we provide some definitions and results from the literature that serve as a background to our main results. In particular, we start by defining risk and variability measures.

**Definition 1** 
(Artzner et al. [44])**.** *A risk measure is a functional $\rho :X\to \bar{R}\equiv \left[-\infty ,+\infty \right]$, where X is a convex cone of risks (random variables) defined on a probability space (Ω,F,P), which may fulfill the following properties:*

*(B0)*  *Law invariance: If X,Y∈X, $X\overset{d}{=}Y$, then ρ(X)=ρ(Y);*
*(B1)*  *Monotonicity: ρ(X)≤ρ(Y) if X≤Y a.s., for X,Y∈X;*
*(B2)*  *Translation invariance: ρ(X+c)=ρ(X)+c for X∈X and any c∈R;*
*(B3)*  *Positive homogeneity: ρ(λX)=λρ(X) for X∈X and any $\lambda \in R_{+}$;*
*(B4)*  *Subadditivity: ρ(X+Y)≤ρ(X)+ρ(Y) for X,Y∈X;*
*(B5)*  *Comonotonic additivity: ρ(X+Y)=ρ(X)+ρ(Y) if X and Y are comonotonic, i.e., there exist two non-decreasing functions f and g such that X=f(Z) and Y=g(Z) almost surely.*

*A risk measure ρ is monetary if it satisfies properties (B1)–(B2), and a risk measure is said to be coherent if it satisfies properties (B1)–(B4).*

**Definition 2** 
(Hu and Chen [13])**.** *A functional $v:X\to {\bar{R}}_{+}\equiv \left[0,\infty \right]$ is a measure of variability if it satisfies law invariance (B0) and the following properties:*

*(C1)*  *Standardization: v(c)=0 for all constants c∈R;*
*(C2)*  *Location invariance: v(X+c)=v(X) for all X∈X,c∈R.*

*If v further satisfies properties (B3) and (B4), it is called a coherent measure of variability.*

**Remark 1.**  *Classical variability measures include variance and the standard deviation. While variance lacks coherence due to its failure to satisfy positive homogeneity (B3) and subadditivity (B4), standard deviation fulfills all coherence requirements. However, neither variance nor standard deviation satisfies comonotonic additivity (B5). For details, see Furman et al. [12].*

We now define the signed Choquet integral, which plays a pivotal role in our theoretical framework.

**Definition 3** 
(Wang et al. [45])**.** *When h:[0,1]→R is of finite variation and such that h(0)=h(1)=0, for all X∈X, the signed Choquet integral is defined by*

(3) $$I\left(X\right)=-\int_{-\infty }^{\infty }h\left(F_{X}\left(x\right)\right)dx,$$

*When h is continuous, Equation (3) can be rewritten as*

(4) $$I\left(X\right)=\int_{0}^{1}F_{X}^{-1}\left(t\right)dh\left(t\right).$$

*Furthermore, when h is absolutely continuous, with dh(t)=ϖ(t)dt, then Equation (4) becomes*

$$I\left(X\right)=\int_{0}^{1}F_{X}^{-1}\left(t\right)\varpi \left(t\right)dt.$$

**Remark 2** 
(Wang et al. [45])**.** *The signed Choquet integral satisfies properties (B0), (B2), (B3), and (B5). Moreover, the functional I, as defined in Equation (3), is subadditive if and only if the distortion function h is convex.*

**Definition 4** 
(Dhaene and Goovaerts [46])**.** *Let $\left(X_{1},X_{2}\right)$ and $\left(Y_{1},Y_{2}\right)$ be random vectors with identical marginals, i.e., $X_{1}\overset{d}{=}Y_{1}$ and $X_{2}\overset{d}{=}Y_{2}$. We say that $\left(X_{1},X_{2}\right)$ are less correlated than $\left(Y_{1},Y_{2}\right)$, and write $\left(X_{1},X_{2}\right){⪯}_{corr}\left(Y_{1},Y_{2}\right)$, if*

(5) $$\mathrm{Cov}\left[h_{1}\left(X_{1}\right),h_{2}\left(X_{2}\right)\right]\leq \mathrm{Cov}\left[h_{1}\left(Y_{1}\right),h_{2}\left(Y_{2}\right)\right]$$

*for all non-decreasing functions $h_{1},h_{2}$ for which the covariances exist.*

*Moreover, if $0<\mathrm{Var}\left[h_{1}\left(X_{1}\right)\right],\;\mathrm{Var}\left[h_{2}\left(X_{2}\right)\right]<\infty$, then*

$$\left(5\right)\;⟺\;\rho \left(h_{1}\left(X_{1}\right),h_{2}\left(X_{2}\right)\right)\leq \rho \left(h_{1}\left(Y_{1}\right),h_{2}\left(Y_{2}\right)\right),$$

*where ρ denotes the Pearson correlation coefficient.*

**Definition 5** 
(Shaked and Shanthikumar [47])**.** *For real-valued random variables X and Y with E[X]=E[Y], we say that X is smaller than Y in convex order, denoted $X{⪯}_{\mathrm{CX}}Y$, if*

E[ϕ(X)]≤E[ϕ(Y)]

*for all increasing convex functions ϕ for which both expectations exist.*

*Under this framework, we have the following property for variability measures*

*(P1)*  *CX-monotonicity: if $X{⪯}_{\mathrm{CX}}Y$, then v(X)≤v(Y).*

In fact, on $L^{q}$, q∈[1,∞], all real-valued coherent measures of variability are CX-monotone (see, e.g., Dana [48] and Föllmer and Schied [43]).

## 3. Shortfall of Tail-Based Entropy Framework

In this section, we develop the shortfall of tail-based entropy (STE) framework in two steps. First, we analyze the properties of tail-based entropy (TE). Second, we construct STE by incorporating TE into expected shortfall (ES) and explore its theoretical properties, showing that STE is coherent under certain conditions.

To ease readability, we have collected the notation used in Section 3 and moved it to the back matter under Nomenclature.

While ES captures tail loss magnitude, it overlooks tail variability. To address this, we introduce TE to measure uncertainty and variability within tail distributions.

For any risk variable *X* and confidence level p∈(0,1), let $X_{p}$ be the tail risk of *X* beyond its *p*-quantile, i.e., $X_{p}\overset{d}{=}F_{X}^{-1}\left(U_{p}\right)$, where $U_{p}$ is a uniform random variable on [p,1]. The distribution function of $X_{p}$ can be represented as

$$F_{X_{p}}\left(x\right)=\left\{\begin{array}{cc} \frac{F_{X}\left(x\right)-p}{1-p}, & \mathrm{when}\;x>x_{p},\\ 0, & \mathrm{otherwise}, \end{array}\right.$$

where $x_{p}=F_{X}^{-1}\left(p\right)$. Thus, for any v∈(0,1), $F_{X_{p}}^{-1}\left(v\right)=F_{X}^{-1}\left(p+(1-p)v\right)$.

Given h∈H, the tail-based entropy (TE) of *X* at confidence level p∈(0,1) is defined by

$$TE_{p}^{h}\left(X\right)=\int_{-\infty }^{\infty }h\left(F_{X_{p}}\left(x\right)\right)\;dx.$$

TE admits both signed Choquet integral and tail covariance representations

(6) $$TE_{p}^{h}\left(X\right)=\int_{0}^{1}F_{X}^{-1}\left(v\right)\;d{\hat{h}}_{p}\left(v\right)=-\mathrm{Cov}\left[X,\varpi \left(\frac{U_{X}-p}{1-p}\right)\;|\;U_{X}>p\right],$$

where ${\hat{h}}_{p}\left(v\right)=0$ for v∈[0,p), and ${\hat{h}}_{p}\left(v\right)=-h\left(\frac{v-p}{1-p}\right)$ for v∈[p,1] and $\varpi =h^{\prime }$. For continuous random variables, (6) reduces to

$$TE_{p}^{h}\left(X\right)=-\mathrm{Cov}\left[X,\varpi \left(\frac{F_{X}\left(X\right)-p}{1-p}\right)\;|\;X>x_{p}\right].$$

The tail covariance representation follows from Zuo and Yin [16]. Moreover, for p=0, TE coincides with the initial entropy (IE): $IE^{h}\left(X\right)=TE_{0}^{h}\left(X\right)$.

**Remark 3.**  *Since TE has the structure of a signed Choquet integral, it satisfies law invariance, translation invariance, positive homogeneity, and comonotonic additivity. Moreover, given that h∈H is concave, IE satisfies subadditivity, thereby establishing IE as a coherent variability measure and ensuring CX-monotonicity.*

**Remark 4.**  *TE measures how tail losses relate to their positions in the distribution through a conditional covariance structure. Unlike variance, which reflects overall variability, TE focuses on the interaction between rank information and loss magnitudes in the tail region. The weighting function h allows practitioners to adjust sensitivity to extremes according to risk preferences.*

To compare tail variability, we adopt the ordering of tail variability introduced by Furman et al. [12], which extends partial orders of variability to tail distributions.

**Definition 6** 
(Furman et al. [12])**.** *For random variable X,Y, we say that Y has a larger p-tail variability compared to X, succinctly $X{⪯}_{p-\mathrm{CX}}Y$, if $F_{X}^{-1}\left(U_{p}\right){⪯}_{\mathrm{CX}}F_{Y}^{-1}\left(U_{p}\right)$.*

Intuitively, the partial order ${⪯}_{p-\mathrm{CX}}$ compares the variability of the two tail distributions $F_{X_{p}}$ and $F_{Y_{p}}$, i.e., the variability of risks beyond the confidence level *p*. However, $ES_{p}$ is not monotone with respect to tail variability, since $ES_{p}\left(X\right)=ES_{p}\left(Y\right)$ whenever $X{⪯}_{p-\mathrm{CX}}Y$ or $Y{⪯}_{p-\mathrm{CX}}X$. In contrast, the following theorem establishes that $TE_{p}^{h}$ is monotone with respect to tail variability.

**Theorem 1.**  *(Proof: see Supplementary Materials (Section S.1.1.)) Let X and Y be real-valued random variables, p∈[0,1), and h∈H. If $X{⪯}_{p-\mathrm{CX}}Y$, then ${\mathrm{TE}}_{p}^{h}\left(X\right)\leq {\mathrm{TE}}_{p}^{h}\left(Y\right)$.*

Next, we define the shortfall of tail-based entropy measure as a linear combination of expected shortfall and tail-based entropy.

**Definition 7.**  *Given h∈H, confidence level p∈[0,1) and loading parameter λ≥0, the shortfall of tail-based entropy (STE) is*

(7) $$STE_{p}^{\lambda ,h}\left(X\right)=ES_{p}\left(X\right)+\lambda \;TE_{p}^{h}\left(X\right),$$

*where $ES_{p}$ and $TE_{p}^{h}$ are defined in (1) and (6), respectively.*

**Remark 5.**  *The parameter λ reflects the decision maker’s aversion to tail uncertainty. For λ=0, STE reduces to ES, reflecting only the mean of tail losses. For λ > 0, STE additionally penalizes tail variability, with larger λ implying greater sensitivity. Thus, λ enables a smooth transition from a pure risk measure to a joint measure of risk and variability.*

The STE framework generalizes various shortfall-type risk measures as special cases through appropriate choices of h∈H, with important examples given in Table 1.

For effective risk management, coherent properties are essential. While the ES is a coherent risk measure, TE is generally not subadditive and, as a variability measure, lacks monotonicity. Consequently, the combined measure STE may fail to retain coherence if λ is too large. When λ=0, STE reduces to the ES and thus inherits coherence; however, for sufficiently large λ, the TE component dominates and may violate coherence. Intuitively, there might be a threshold that delineates the value of λ for which STE is coherent. As we show in the next theorem, the thresholds for both monotonicity and sub-additivity are the same and equal to $\frac{1}{\varpi (0)}$ with ϖ(0)≠0.

**Theorem 2.**  *(Proof: see Supplementary Materials (Section S.1.2.)) Given h∈H, confidence level p∈[0,1) and loading parameter λ≥0.*

*(1)*  *$STE_{p}^{\lambda ,h}$ admits the following representations:*
$$\begin{array}{cc} STE_{p}^{\lambda ,h}\left(X\right)= & \int_{0}^{1}F_{X}^{-1}\left(u\right)d{\hat{h}}_{p}^{\lambda }\left(u\right)\\ = & \int_{0}^{1}F_{X}^{-1}\left(u\right)\gamma_{p}^{\lambda }\left(u\right)du\\ = & E[X\gamma_{p}^{\lambda }\left(U_{X}\right)], \end{array}$$
  *where $\varpi =h^{\prime }$, with ${\hat{h}}_{p}^{\lambda }\left(u\right)$ and $\gamma_{p}^{\lambda }\left(u\right)$ defined by*
$${\hat{h}}_{p}^{\lambda }\left(u\right)=\left[\frac{u-p}{1-p}-\lambda h\left(\frac{u-p}{1-p}\right)\right]I_{\left[p,1\right]}\left(u\right),$$
  *and*
$$\gamma_{p}^{\lambda }\left(u\right)=\left[\frac{1}{1-p}-\frac{\lambda }{1-p}\varpi \left(\frac{u-p}{1-p}\right)\right]I_{\left[p,1\right]}\left(u\right).$$
*(2)*  *$STE_{p}^{\lambda ,h}\left(X\right)$ is law invariant, translation invariant, positive homogeneous and comonotonically additive.*
*(3)*  *The following conditions are equivalent:*
  *(a)*  *$STE_{p}^{\lambda ,h}\left(X\right)$ is monotone;*
  *(b)*  *$STE_{p}^{\lambda ,h}\left(X\right)$ is subadditive;*
  *(c)*  *$STE_{p}^{\lambda ,h}\left(X\right)$ holds the increasing convex order;*
  *(d)*  *$STE_{p}^{\lambda ,h}\left(X\right)$ is a coherent risk measure;*
  *(e)*  *$\lambda \in \left[0,\frac{1}{\varpi (0)}\right],\;\;\varpi \left(0\right)\neq 0$.*

**Remark 6.**  *Although λ can take any non-negative value mathematically, Theorem 2 requires λ∈[0,1/ϖ(0)] (with ϖ(0)≠0) to ensure coherence. The penalty will become too harsh near the quantile when λ is excessively large, which can lead to violation of subadditivity; this is why λ must be bounded by 1/ϖ(0). In practice, λ may be determined based on regulatory requirements, historical data calibration, utility functions, or institutional risk tolerance. It can then be adjusted dynamically according to macroeconomic conditions and market dynamics. The optimal choice of λ depends on the emphasis placed on tail variability: highly risk-averse institutions select larger λ to impose stronger penalties on tail uncertainty, while applications relatively insensitive to tail fluctuations prefer smaller λ.*

Since comonotonically additive and coherent risk measures are known as spectral risk measures, STE belongs to this class when λ satisfies the coherence condition. Consequently, STE-based capital allocation can be embedded into established frameworks for coherent risk measure allocation (see, e.g., Cherny [49], Cherny and Orlov [50], Tsanakas [51]). Compared to other spectral measures, STE has three distinctive features. First, it decomposes tail risk into magnitude (ES) and variability (TE), providing interpretable components. Second, it respects tail variability ordering (Theorem 1), unlike ES. Third, the parameter λ allows for flexible calibration to different risk preferences.

## 4. STE-Based Allocation

This section develops capital allocation rules based on the STE risk measure. We define the specific form of STE-based allocation, reveal its structural characteristics that simultaneously reflect tail expectation and variability, and analyze its fundamental properties. Under the centered regression assumption, we derive closed-form allocation expressions. We also establish the connection between STE allocation and the distance-minimization allocation framework proposed by Dhaene et al. [23], which has been widely applied (e.g., Cai and Wang [52]; Wang et al. [53]).

Motivated by capital adequacy requirements in modern financial regulation and exemplified by Solvency II and Basel III frameworks (Sandström [54]; Cannata and Quagliariello [55]), here, we introduce a capital allocation counterpart to the shortfall of tail-based entropy (STE).

Consider a portfolio $X=\left(X_{1},\ldots ,X_{n}\right)\in X^{n}$ with aggregate loss $S=\sum_{i=1}^{n}X_{i}$. Given a total capital *K*, an allocation $\left(K_{1},\ldots ,K_{n}\right)$ satisfies $K=\sum_{i=1}^{n}K_{i}$. Let $U_{S}$ denote the distributional transform of *S* (Proposition 1.3 of Rüschendorf [56])

$$U_{S}=F_{S}\left(S-\right)+V\left(F_{S}\left(S\right)-F_{S}\left(S-\right)\right),\;V∼U\left[0,1\right]\;\mathrm{independent}\;\mathrm{of}\;\left(X_{1},\ldots ,X_{n}\right),$$

where F(y−) denotes the left-hand limit. Then $U_{S}∼U\left[0,1\right]$ and $F_{S}^{-1}\left(U_{S}\right)=S$ almost surely.

**Definition 8.**  *Given h∈H, confidence level p∈(0,1) and loading parameter λ≥0, the STE-based allocation is defined by*

(8) $$\begin{array}{c} STE_{p}^{\lambda ,h}\left(X_{i},S\right)=ES_{p}\left(X_{i},S\right)+\lambda \;TE_{p}^{h}\left(X_{i},S\right), \end{array}$$

*where $ES_{p}\left(X_{i},S\right)=E\left[X_{i}|U_{S}>p\right]$ and $TE_{p}^{h}\left(X_{i},S\right)=-\mathrm{Cov}\left[X_{i},\varpi \left(\frac{U_{S}-p}{1-p}\right)|U_{S}>p\right]$.*

If $P(S=s_{p})=0$ with $s_{p}:=F_{S}^{-1}\left(p\right)$, then (8) reduces to

(9) $$STE_{p}^{\lambda ,h}\left(X_{i},S\right)=E\left[X_{i}|S>s_{p}\right]-\lambda \;\mathrm{Cov}\left[X_{i},\varpi \;\left(\frac{F_{S}\left(S\right)-p}{1-p}\right)\;|\;S>s_{p}\right].$$

Note the obvious though important equation $\sum_{i=1}^{n}STE_{p}^{\lambda ,h}\left(X_{i},S\right)=STE_{p}^{\lambda ,h}\left(S,S\right)$, with the right-hand side equal to $STE_{p}^{\lambda ,h}\left(S\right)$ given by (7) with the aggregate risk *S* in the role of *X*.

Denote the capital allocation proportion to the business *i* by $k_{i}$, then

$$k_{i}=\frac{STE_{p}^{\lambda ,h}\left(X_{i},S\right)}{STE_{p}^{\lambda ,h}\left(S\right)}=\frac{ES_{p}\left(X_{i},S\right)+\lambda \;TE_{p}^{h}\left(X_{i},S\right)}{ES_{p}\left(S\right)+\lambda \;TE_{p}^{h}\left(S\right)}.$$

**Remark 7.**  *When appropriate regularity conditions (see Proposition 1 of Tsanakas and Millossovich [57]) are satisfied for the joint distribution of $\left(X_{1},\ldots ,X_{n},S\right)$, our proposed STE-based allocation rules in Equation (8) coincides with the Euler allocation principles (see Section 8.5 of McNeil et al. [58]).*

Some elementary properties are given by Proposition 1.

**Proposition 1.**  *(Proof: see Supplementary Materials (Section S.2.2.)) Given h∈H, confidence level p∈(0,1) and loading parameter λ≥0, the STE-based allocation satisfies the following properties:*

*1*.  *For i=1,…,n*
$$STE_{p}^{\lambda ,h}\left(X_{i},S\right)\geq 0\Leftrightarrow \mathrm{Cov}\left[X_{i},\gamma_{p}^{\lambda }\left(U_{S}\right)\right]\geq -E\left[X_{i}\right].$$
*2*.  *If $X=(X_{1},\ldots ,X_{n})$ has continuous marginals and is comonotonic*
$$STE_{p}^{\lambda ,h}\left(X_{i},S\right)=STE_{p}^{\lambda ,h}\left(X_{i}\right).$$
*3*.  *For i=1,…,n and for $\lambda \in [0,\frac{1}{\varpi (0)}]$ with ϖ(0)≠0*
$$STE_{p}^{\lambda ,h}\left(X_{i},S\right)\leq STE_{p}^{\lambda ,h}\left(X_{i}\right).$$
*4*.  *Suppose $X_{i}\overset{d}{=}X_{j}$, and $\left(X_{i},S\right){⪯}_{corr}\left(X_{j},S\right)$ for all i,j=1,…,n, and let $\lambda \in [0,\frac{1}{\varpi (0)}]$ with ϖ(0)≠0, then*
$$STE_{p}^{\lambda ,h}\left(X_{i},S\right)\leq STE_{p}^{\lambda ,h}\left(X_{j},S\right).$$

Part (1) provides a criterion for the sign of capital allocation. A unit acts as a risk contributor (positive allocation) if its tail scenario weighted comovement with the portfolio is sufficiently large to compensate for its expected loss. Otherwise, it acts as a risk mitigator (negative allocation). Part (2) characterizes the capital allocation boundary under the strongest form of positive dependence. In this setting, diversification effects are fully exhausted, and each unit’s allocated capital equals its stand-alone capital requirement. Part (3) states that the STE-based allocation is individually rational and reflects diversification. Within the coherence range, each unit’s allocation is no greater than its stand-alone requirement, and the resulting capital relief measures its marginal contribution to portfolio risk reduction. Part (4) reflects the dependence monotonicity of the STE-based allocation. Under identical marginals and within the coherence range, units that are more concordant with the portfolio (i.e., larger in the correlation order) receive larger capital allocations, as stronger tail comovement reduces diversification.

Furman and Zitikis [27] showed that, under a linear centered regression structure, risk allocation admits a CAPM-style linear form. Following this idea, we incorporate the same assumption into the STE framework—a composite measure capturing tail variability to obtain a closed-form and interpretable allocation formula.

**Theorem 3.**  *(Proof: see Supplementary Materials (Section S.2.3.)) Assume that the centered regression function $\gamma_{X_{i}|S}\left(s\right)=E\left[X_{i}-E\left[X_{i}\right]|S=s\right]$ can be written as*

(10) $$\gamma_{X_{i}|S}=C\left(F_{X_{i},S}\right)\left(s-E\left[S\right]\right),$$

*with some $C(F_{X_{i},S})$ that does not depend on the function h and parameter λ. Then*

(11) $$STE_{p}^{\lambda ,h}\left(X_{i},S\right)=E\left[X_{i}\right]+C\left(F_{X_{i},S}\right)\left(STE_{p}^{\lambda ,h}\left(S\right)-E\left(S\right)\right).$$

**Remark 8.**  *The centered regression assumption in (10) holds in important model classes, including elliptically contoured distributions and certain background risk factor constructions (e.g., the classical bivariate Pareto under multiplicative background risk and joint models with gamma margins under additive/multiplicative background risk; see Furman et al. [33]).*

*Under this assumption, Theorem 3 delivers a CAPM-style linear representation of the STE-based allocation: an expected loss baseline plus a systematic tail risk premium, with the TE component imposing rank-dependent weights (via h) on tail scenarios. The sensitivity coefficient $C(F_{X_{i},S})$ depends only on the joint distribution of $\left(X_{i},S\right)$, and is thus independent of h and λ, quantifying the unit’s marginal exposure to systematic tail risk.*

We now investigate the connection between the STE-based allocation and the optimal capital allocation framework of Dhaene et al. [23], which is formulated as the following distance-minimization problem:

(12) $$\min_{K\in A}\sum_{i=1}^{n}v_{i}\;E\left[\zeta_{i}D\left(\frac{X_{i}-K_{i}}{v_{i}}\right)\right],$$

where $K\in A:=\left\{K\in R^{n}:\sum_{i=1}^{n}K_{i}=K\right\}$, $v_{i}\geq 0$ with $\sum_{i=1}^{n}v_{i}=1$, $\zeta_{i}\geq 0$ are random variables satisfying $E[\zeta_{i}]=1$, and *D* denotes a distance measure.

**Proposition 2.**  *(Proof: see Supplementary Materials (Section S.2.4.)) Let p∈(0,1) and $\lambda \in \left[0,\frac{1}{\varpi (0)}\right]$ with ϖ(0)≠0. Then, the three characterizing elements required to represent the STE-based allocation principle in the general framework defined by (12) are*

*1*.  *$D\left(x\right)=x^{2}$,*
*2*.  *$v_{i}=\frac{E[\zeta_{i}X_{i}]}{\sum_{j=1}^{n}E\left[\zeta_{j}X_{j}\right]},\;i=1,\ldots ,n$,*
*3*.  *$\zeta_{i}=\gamma_{p}^{\lambda }\left(U_{S}\right)$, i=1,…,n.*

## 5. STE-Based Allocation for Some Parametric Distribution

This section derives explicit allocation formulas for STE measures under two important distribution families: elliptical distributions and extended skew-normal distributions. These families are chosen for their prevalence in financial modeling and their ability to capture both symmetric and asymmetric tail behaviors crucial for risk management.

In Section 5.1 and Section 5.2 (non-degenerate elliptical and extended skew-normal settings), *S* has a continuous distribution, hence $P(S=s_{p})=0$ with $s_{p}:=F_{S}^{-1}\left(p\right)$; accordingly, the STE-based allocation is given by (9).

### 5.1. Elliptical Distribution

**Definition 9.**
  *A random vector $X={\left(X_{1},\ldots ,X_{n}\right)}^{T}$ follows an elliptical distribution with location parameter $\mu ={\left(\mu_{1},\ldots ,\mu_{n}\right)}^{T}$ and positive definite scale matrix **Σ**, denoted $X∼E_{n}\left(\mu ,\Sigma ,g_{n}\right)$, if its density function (when it exists) has the form*

(13) $$f_{X}\left(x\right)=\frac{c_{n}}{\sqrt{\left|\Sigma \right|}}g_{n}\left[\frac{1}{2}{\left(x-\mu \right)}^{T}\Sigma^{-1}\left(x-\mu \right)\right],$$

*where*

$$c_{n}=\frac{\Gamma (n/2)}{{\left(2\pi \right)}^{n/2}}{\left[\int_{0}^{\infty }x^{n/2-1}g_{n}\left(x\right)dx\right]}^{-1},$$

*and*

$$\int_{0}^{\infty }x^{n/2-1}g_{n}\left(x\right)dx<\infty ,$$

*which guarantees $g_{n}\left(x\right)$ to be the density generator.*

Consider a univariate elliptically distributed random variable $X∼E_{1}\left(\mu ,\sigma^{2},g_{1}\right)$, that is X=μ+σZ, where $Z∼S(g_{1})$ is a spherical random variable with density function $f_{Z}\left(z\right)=cg_{1}\left(\frac{z^{2}}{2}\right)$, and the tail generator function is defined as $\bar{G}\left(z\right)=c\int_{z}^{\infty }g_{1}\left(u\right)du$.

For elliptical distributions $X∼E_{n}\left(\mu ,\Sigma ,g_{n}\right)$ with aggregate risk $S=\sum_{i=1}^{n}X_{i}$, we have $S∼E_{1}\left(\mu_{S},\sigma_{S}^{2},g_{1}\right)$ where $\mu_{S}=\sum_{j=1}^{n}\mu_{j}$, $\sigma_{S}^{2}=\sum_{j=1}^{n}\sum_{k=1}^{n}\sigma_{jk}$, $\sigma_{i,S}=\sum_{j=1}^{n}\sigma_{ij}$, and the regression result $E\left[X_{i}|S=s\right]=\mu_{i}+\frac{\sigma_{i,S}}{\sigma_{S}^{2}}\left(s-\mu_{S}\right)$.

The following theorem provides the closed-form capital allocation formula under the STE measure when the loss vector follows an elliptical distribution.

**Theorem 4.**  *(Proof: see Supplementary Materials (Section S.3.2.)) Consider $X∼E_{n}\left(\mu ,\Sigma ,g_{n}\right)$ with finite mean and covariance, under the assumptions that h∈H is twice differentiable, and ϖ(0) exists. Then for every p∈(0,1) and i=1,…,n, we have*

(14) $$\begin{array}{cc} STE_{p}^{\lambda ,h}\left(X_{i},S\right) & =\mu_{i}+\frac{\sigma_{i,S}}{\sigma_{S}}\left(\left(1-\lambda \varpi (0)\right)ES_{p}\left(Z\right)\right.\\ \; & \;\left.-\frac{\lambda }{1-p}E\left[\varpi^{\prime }\left(\frac{F_{Z}\left(Z\right)-p}{1-p}\right)\bar{G}\left(Z^{2}/2\right)|Z>z_{p}\right]\right). \end{array}$$

*Moreover, if Var(Z)<∞, Equation (14) admits an equivalent representation*

$$\begin{array}{cc} STE_{p}^{\lambda ,h}\left(X_{i},S\right) & =\mu_{i}+\frac{\sigma_{i,S}}{\sigma_{S}}(\left(1-\lambda \varpi \left(0\right)\right)\;ES_{p}\left(Z\right)\\ \; & \;-\frac{\lambda \sigma_{Z}^{2}}{1-p}\;E\left[\varpi^{\prime }\left(\frac{F_{Z}\left(Z\right)-p}{1-p}\right)f_{Z^{*}}\left(Z\right)|Z>z_{p}\right]), \end{array}$$

where $z_{p}=F_{Z}^{-1}\left(p\right)$, $ES_{p}\left(Z\right)=\frac{\bar{G}\left(z_{p}^{2}/2\right)}{1-p}$ and $f_{Z^{*}}\left(z\right)=\frac{1}{\sigma_{Z}^{2}}\bar{G}\left(z^{2}/2\right)$ is the density of a spherical random variable $Z^{*}$.

**Remark 9.**
  *Under elliptical distributions, since the conditional expectation has a linear regression structure, the STE-based allocation is formally similar to the traditional variance–covariance allocation. However, the portfolio’s aggregate risk is measured by STE, and thus the two are not informationally equivalent. By employing rank-dependent tail weighting, STE characterizes the structure of the tail distribution. It captures aspects that traditional second-moment methods overlook, enhancing sensitivity to tail thickness, extreme outcomes, and systemic risk. When the conditional structure deviates from linearity (e.g., in extended skew-normal distributions), the STE allocation formula naturally incorporates nonlinear corrections driven by skewness and higher-order dependence, thereby yielding an allocation structure that goes beyond the traditional variance–covariance framework. The extended skew-normal distribution will be used as an example in the sequel.*

From Theorem 4, we obtain closed-form capital allocation expressions for various STE measures under elliptical distributions, which are summarized in Table 2.

### 5.2. Extended Skew-Normal Distribution

The extended skew normal distribution according to Capitanio et al. [59] is an extension of the normal distributions and is useful in modelling the data presenting skewness.

**Definition 10.**
  *A continuous n-dimensional random vector X is said to have a multivariate extended skew normal distribution with location vector $\mu \in R^{n}$, positive definite n×n dispersion matrix* **Σ** *, shape parameter $\alpha \in R^{n}$, extension parameter τ∈R, if its density is given by*

$$f_{X}\left(x\right)=\frac{1}{\Phi (\tau /c)}{\left|\Sigma \right|}^{-\frac{1}{2}}\Phi \left(\tau +\alpha^{T}\left(x-\mu \right)\right)\phi_{n}\left(x;\mu ,\Sigma \right),$$

*where $c={\left(1+\alpha^{T}\Sigma \alpha \right)}^{1/2}$, $\alpha =\Sigma^{-1}\delta {\left(1-\delta^{T}\Sigma^{-1}\delta \right)}^{-\frac{1}{2}}$, $\phi_{n}\left(\cdot ;\mu ,\Sigma \right)$ is the n-dimensional normal density function, and Φ(·) is the standard normal distribution function. In this case, we shall write $X∼ESN_{n}\left(\mu ,\Sigma ,\tau ,\alpha \right)$.*

We recall that the multivariate extended skew-normal family is closed under affine transformations (see Li and Yin [42]). Specifically, if $X∼ESN_{n}\left(\mu ,\Sigma ,\tau ,\alpha \right)$, then, for any m×n matrix *A* of rank m≤n and m×1 vector b, it holds that $AX+b∼ESN_{m}\left(\mu_{A},\Sigma_{A},\tau_{A},\alpha_{A}\right)$, where $\mu_{A}=A\mu +b$, $\kappa_{A}={\left[1+\alpha^{T}\left(\Sigma -\Sigma A^{T}\Sigma_{A}^{-1}A\Sigma \right)\alpha \right]}^{\frac{1}{2}}$, $\Sigma_{A}=A\Sigma A^{T}$, ${\bar{\alpha }}_{A}=\Sigma_{A}^{-1}A\Sigma \alpha$, $\alpha_{A}=\kappa_{A}^{-1}{\bar{\alpha }}_{A}$ and $\tau_{A}=\kappa_{A}^{-1}\tau$.

In particular, with $e={\left(1,\ldots ,1\right)}^{⊤}\in R^{n}$ set $S=e^{⊤}X$ and $Z=\left(S-\mu_{S}\right)/\sigma_{S}$. Consequently, $S∼ESN_{1}\left(\mu_{S},\sigma_{S}^{2},\tau_{S},\alpha_{S}\right)$ and $Z∼ESN_{1}\left(0,1,\tau_{Z},\alpha_{Z}\right)$, where $\mu_{S}=e^{T}\mu$, $\sigma_{S}^{2}=e^{⊤}\Sigma e$, $\kappa_{S}={\left[1+\alpha^{⊤}\left(\Sigma -\sigma_{S}^{-2}\Sigma ee^{⊤}\Sigma \right)\alpha \right]}^{1/2}$, $\alpha_{S}=\kappa_{S}^{-1}\sigma_{S}^{-2}e^{⊤}\Sigma \alpha$, $\tau_{S}=\kappa_{S}^{-1}\tau$, and $\tau_{Z}=\tau_{S}$, $\alpha_{Z}=\sigma_{S}\alpha_{S}$.

Contrasting the elliptical case, the ESN setting admits a linear term plus a skewness-driven deviation in $E[X_{i}|S=s]$, we state the precise form below.

**Lemma 1.**  *(Proof: see Supplementary Materials (Section S.3.3.)) Let $X∼ESN_{n}\left(\mu ,\Sigma ,\tau ,\alpha \right)$. Then*

(15) $$E\left[X_{i}|S=s\right]=s\frac{\sigma_{i,S}}{\sigma_{S}^{2}}+\delta_{i.S}\eta_{i.S}\left(s\right)+k_{i},$$

where $\sigma_{ii.S}=\sigma_{i}^{2}-{\left(\sigma_{i,S}/\sigma_{S}\right)}^{2}$, $\delta_{i.S}={\left(1+{\left(\alpha_{i}^{*}\right)}^{2}\sigma_{ii.S}\right)}^{-1/2}\sigma_{ii.S}\alpha_{i}^{*}$, $k_{i}=\mu_{i}-\mu_{S}\left(\sigma_{i,S}/\sigma_{S}^{2}\right)$ and $\eta_{i.S}\left(s\right)=\phi \left(\frac{\tau_{i.S}\left(s\right)}{c_{i.S}}\right)/\Phi \left(\frac{\tau_{i.S}\left(s\right)}{c_{i.S}}\right)$. Here $c_{i,S}={\left(1+{\left(\alpha_{i}^{*}\right)}^{2}\sigma_{ii.S}\right)}^{1/2}$ and $\tau_{i.S}\left(s\right)=\tau_{i,S}+\left(\alpha_{S}^{*}+\sigma_{S}^{-2}\sigma_{i,S}\alpha_{i}^{*}\right)\left(s-\mu_{S}\right)$.

The following theorem provides the closed-form capital allocation formula under the STE measure when the loss vector follows an extended skew-normal distribution.

**Theorem 5.**  *(Proof: see Supplementary Materials (Section S.3.5.)) Consider $X∼ESN_{n}\left(\mu ,\Sigma ,\tau ,\alpha \right)$ with finite mean and covariance, under the assumptions that h∈H is twice differentiable, and ϖ(0), ϖ(1) both exist. Then for every p∈(0,1) and i=1,…,n, we have*

$$\begin{array}{cc} STE_{p}^{\lambda ,h}\left(X_{i},S\right)= & \mu_{i}+\delta_{i.S}E\left[\eta_{i.S}\left(S\right)\left(1-\lambda \;\varpi \left(\frac{F_{S}\left(S\right)-p}{1-p}\right)\right)|S>s_{p}\right]\\ + & \frac{\sigma_{i,S}}{\sigma_{S}}\left(ES_{p}\left(Z\right)+\lambda \;TE_{p}^{h}\left(Z\right)\right). \end{array}$$

*where $s_{p}=F_{S}^{-1}\left(p\right)$, $z_{p}=F_{Z}^{-1}\left(p\right)$,*

$$ES_{p}\left(Z\right)=\frac{1}{1-p}\left(f_{Z}\left(z_{p}\right)+\frac{\alpha_{Z}\eta_{Z}}{c_{Z}}\bar{\Phi }\left(c_{Z}z_{p}+\frac{\tau_{Z}\alpha_{Z}}{c_{Z}}\right)\right),$$

*and*

$$\begin{array}{cc} TE_{p}^{h}\left(Z\right)= & -\frac{1}{1-p}\left[E\left[\left(f_{Z}\left(Z\right)+\frac{\alpha_{Z}\eta_{Z}}{c_{Z}}\Phi \left(Zc_{Z}+\frac{\tau_{Z}\alpha_{Z}}{c_{Z}}\right)\right)\varpi^{\prime }\left(\frac{F_{Z}\left(Z\right)-p}{1-p}\right)|Z>z_{p}\right]\right.\\ \; & +\left.\frac{\alpha_{Z}\eta_{Z}}{c_{Z}}\left(\varpi (1)-\varpi (0)\right)\right]-\varpi \left(0\right)ES_{p}\left(Z\right). \end{array}$$

**Remark 10.**
  *Theorem 5 shows that under the extended skew-normal distribution the STE-based allocation departs from the linear covariance form and incorporates nonlinear correction terms driven by skewness. These terms arise from the deviation component $\delta_{i.S}\eta_{i,S}\left(S\right)$ in the marginal conditional expectation. Through its interaction with the tail-weighting function ϖ(·), the allocation becomes responsive to tail skewness and extreme value behavior. Thus STE extends covariance-based allocation to settings where conditional expectations are non-linear.*

From Theorem 5, we obtain closed-form capital allocation expressions for various STE measures under extended skew-normal distributions, which are summarized in Table 3.

### 5.3. Discussion

**Figure 1:**
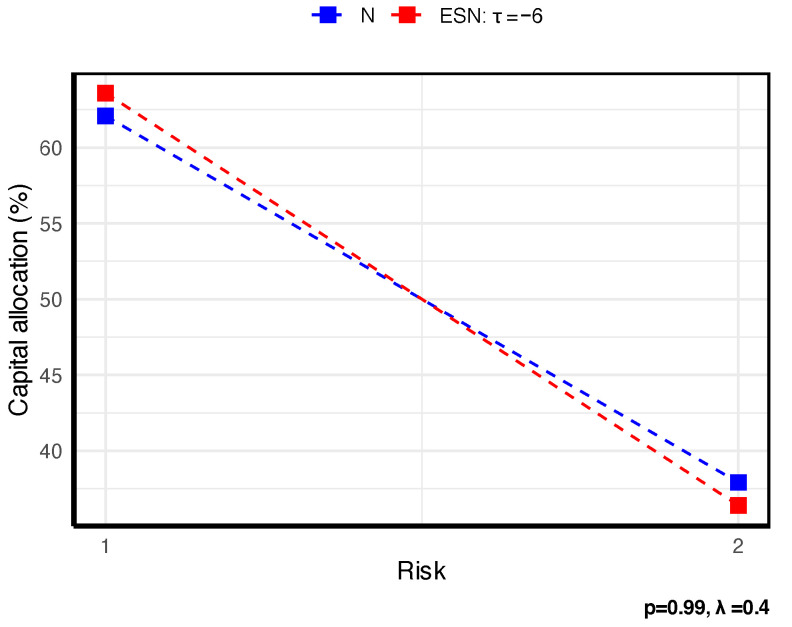
Illustration of the impact of dependence on STE-based allocation.

**Figure 2:**
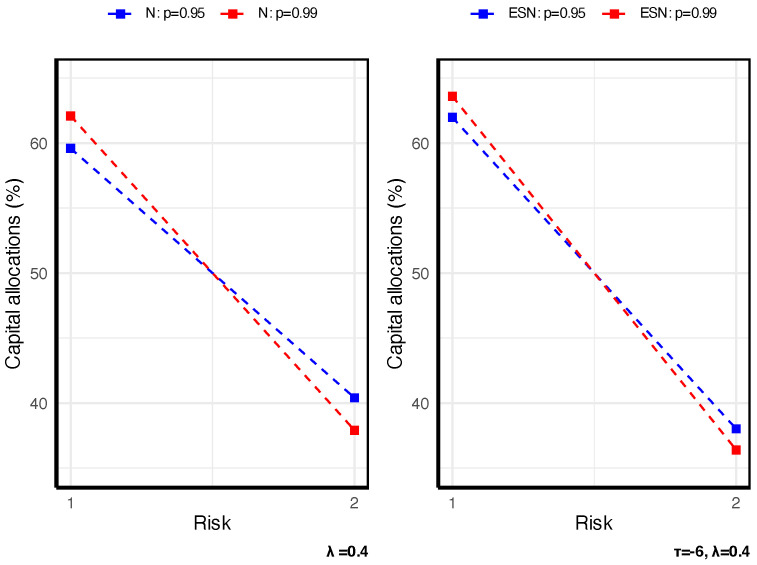
Illustration of the impact of risk level on STE-based allocation.

**Figure 3:**
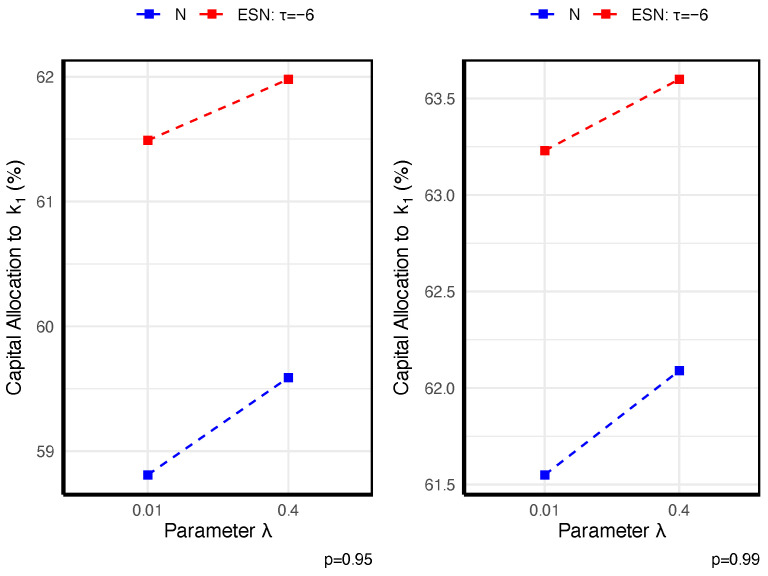
Illustration of the impact of parameter λ on STE-based allocation.

To illustrate how STE-based allocations react under different conditions, we present a concise set of graphical examples based on two fixed bivariate models. First, consider two business lines $X={\left(X_{1},X_{2}\right)}^{⊤}∼N_{2}\left(\mu ,\Sigma \right)$ with $\mu ={\left(2,4\right)}^{⊤}$ and $\Sigma =\left(\begin{array}{cc} 10 & 1\\ 1 & 2 \end{array}\right)$. For comparison, we also analyze $Y={\left(Y_{1},Y_{2}\right)}^{⊤}∼ESN_{2}\left(\mu ,\Sigma ,\tau ,\alpha \right)$ with $\alpha ={\left(1,3\right)}^{⊤}$, where τ controls skewness and one-sided tail concentration. Throughout, we allocate capital using the Gini shortfall (GS), a special case of STE, and we summarize how the allocations vary with the dependence structure, the penalty parameter λ, and the tail level *p*. All results are presented graphically in Figure 1, Figure 2 and Figure 3.

Figure 1 shows that stronger dependence intensifies tail comovement, thereby allocating more capital to ($X_{1}\left(Y_{1}\right)$), which is more concordant with the aggregate loss. Figure 2 shows that as the tail level *p* increases, the insurer adopts a more conservative risk stance, leading to a shift of capital toward the riskier business line. Figure 3 shows that, across all specifications, the capital allocated to the riskiest line—$X_{1}$ ($Y_{1}$)—increases with the penalty parameter λ, which reflects the penalty λ on the TE of the new model, as expected. Figure 3 further indicates that, relative to $X_{1}$, the higher skewness of $Y_{1}$ entails a larger capital allocation. Hence, in practice, one should always seek a suitably skewed distribution if the faced risks are skewed.

## 6. Applications

In this section, we investigate the STE-based allocation rules using an insurance dataset originally introduced by Panjer [60]. This dataset has been extensively employed in capital allocation studies, see, e.g., Xu and Mao [29], Xu [30], and Furman et al. [12]. Specifically, we focus on two key special cases: the extended Gini shortfall ($EGS_{r,p}^{\lambda }$) and the cumulative residual entropy shortfall ($CRES_{p}^{\lambda }$).

In the following, we give the definition of a multivariate Student-*t* distribution.

**Definition 11.**  *The n-dimensional multivariate Student-t distribution can be characterized as a member of the elliptical family through its density generator, which takes the form*

$$g_{n}\left(x\right)={\left(1+\frac{x}{c_{q}}\right)}^{-q},$$

*where q>n/2, and $c_{q}$ represents a parameter depending on q. For simplicity, we assume that q=n+ν with degrees of freedom ν and $c_{n}=\nu /2$, the corresponding probability density function is given by:*

$$f\left(x\right)=\frac{c_{n}}{\sqrt{\left|\Sigma \right|}}{\left[1+\frac{{\left(x-\mu \right)}^{T}\Sigma^{-1}\left(x-\mu \right)}{\nu }\right]}^{-(n+\nu )/2},$$

*with the normalizing constant $c_{n}=\frac{\Gamma ((n+\nu )/2)}{\Gamma (\nu /2)}{\left(\pi \nu \right)}^{-n/2}$.*

The dataset comprises the present values of required capital reserves to ensure solvency over a specified time horizon at high confidence levels. While Panjer [60] assumed a multivariate normal distribution for the joint distribution of these variables, we instead assume a multivariate Student-*t* distribution with a specified mean vector

$$\mu ={\left(25.69,37.84,0.85,12.70,0.15,24.05,14.41,4.49,4.39,9.56\right)}^{T}$$

and covariance matrix

$$\Sigma =\left(\begin{array}{cccccccccc} 7.24 & 0 & 0.07 & -0.07 & 0.28 & -2.71 & -0.51 & 0.28 & 0.23 & -0.21\\ 0 & 20.16 & 0.05 & 1.60 & 0.05 & 1.39 & 1.14 & -0.91 & -0.81 & -1.74\\ 0.07 & 0.05 & 0.04 & 0.00 & -0.01 & 0.08 & 0.01 & -0.02 & -0.02 & -0.07\\ -0.07 & 1.60 & 0.00 & 1.12 & 0.17 & 0.26 & 0.19 & -0.14 & 0.18 & -0.79\\ 0.28 & 0.05 & -0.01 & 0.17 & 0.32 & -0.24 & 0.01 & -0.02 & 0.08 & -0.01\\ -2.71 & 1.39 & 0.08 & 0.26 & -0.24 & 14.98 & 0.43 & -0.33 & -1.89 & -1.60\\ -0.51 & 1.14 & 0.01 & 0.19 & 0.01 & 0.43 & 2.53 & -0.38 & 0.13 & 0.58\\ 0.28 & -0.91 & -0.02 & -0.14 & -0.02 & -0.33 & -0.38 & 0.92 & -0.16 & -0.40\\ 0.23 & -0.81 & -0.02 & 0.18 & 0.08 & -1.89 & 0.13 & -0.16 & 1.12 & 0.58\\ -0.21 & -1.74 & -0.07 & -0.79 & -0.01 & -1.60 & 0.58 & -0.40 & 0.58 & 6.71 \end{array}\right)$$

The Pearson correlations of the risk due to the *k*th business line and the aggregate risk *S* are

$$\rho_{k,s}={\left(0.25,0.69,0.09,0.36,0.16,0.40,0.39,-0.18,-0.07,0.18\right)}^{T}.$$

To study the portfolio diversification effects, we define the diversification per unit of risk (DIV) as

$$DIV=1-\frac{\rho (S)}{\sum_{i=1}^{n}\rho \left(X_{i}\right)},$$

where ρ(S) is the portfolio risk and $\sum_{i=1}^{n}\rho \left(X_{i}\right)$ is the sum of stand-alone risks. Higher values indicate greater diversification benefits.

We now analyze DIV under parameters λ∈{0.5,1}, ν∈{2,5,∞}, and p=0.75, with the results summarized in Table 4. In the heavy-tailed case, ${\mathrm{STE}}_{p}^{\lambda ,h}$ measures exhibit superior diversification performance relative to ${\mathrm{ES}}_{p}$. For the case of (ν,λ)=(2,0.5), the DIV of ${\mathrm{EGS}}_{1.4,p}^{\lambda }$ is 23.81%, significantly higher than the 17.01% of ${\mathrm{ES}}_{p}$; increasing λ to 1.0 further raises it to 28.93%, indicating that higher tail sensitivity improves the detection of potential risk-reduction benefits. This difference aries from their distinct characterizations of tail structure. While ${\mathrm{ES}}_{p}$ focuses solely on the tail conditional mean, STE, through the ${\mathrm{TE}}_{p}^{h}$ component, also captures the volatility and comovement structure within the tail. This allows for it to better identify partial hedging among risks in extreme scenarios, thereby exhibiting higher diversification effects. As the distribution approaches light tails, this advantage gradually diminishes. In contrast, ${\mathrm{SDS}}_{p}^{\lambda }$ fails under heavy tails due to the absence of second moments, underscoring the robustness of ${\mathrm{STE}}_{p}^{\lambda ,h}$ measures in extreme risk settings.

**Figure 4:**
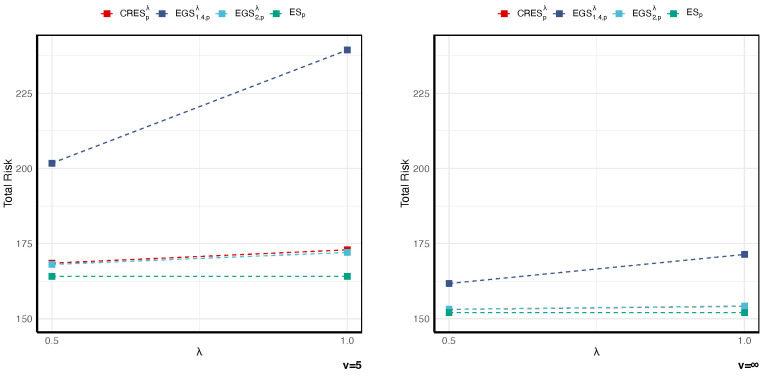
Total risk comparison of ES, EGS, and CRES for varying *v*, λ, as well as p=0.99.

**Figure 5:**
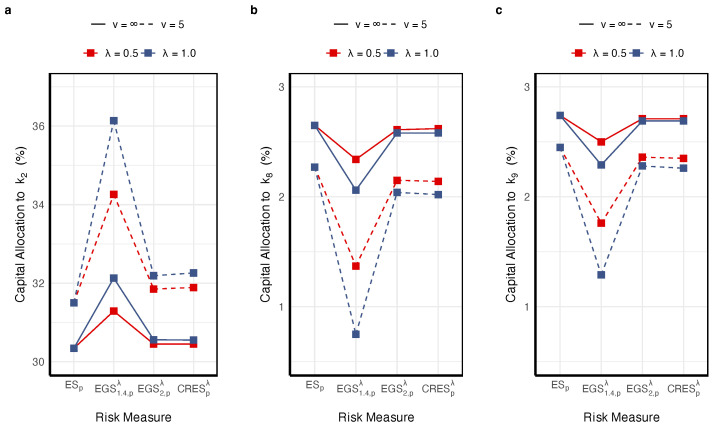
Capital allocation percentage comparison for different risks (**a**) $X_{2},\left(b\right)\;X_{8},\left(c\right)\;X_{9}$.

To further demonstrate the advantages of the STE framework, we analyze the capital allocation for a portfolio of ten business lines, as presented in Table 5 and Figure 4 and Figure 5.

As shown in Figure 4 and Table 5, under heavy-tailed distributions, STE-based methods require substantially higher capital than traditional risk measures. For example, when (ν,λ)=(5,1), $EGS_{1.4,p}^{\lambda }$ equals 239.424, whereas $ES_{p}$ is 164.084. This difference is not a weakness, but rather the intended design of the framework: the additional capital acts as a variability buffer that reflects the highly uncertain, heavy-tailed risks of the portfolio—risks that $ES_{p}$ completely ignores. As tails become lighter (ν→∞), the risk penalty of the TE component diminishes, and the differences between STE and ES converge, validating the theoretical consistency of the framework. From a risk management perspective, the higher capital requirement under STE stems from measuring tail variability rather than from undue conservatism. The parameter λ captures the institution’s risk appetite toward tail uncertainty. It allows for the capital buffer to adjust adaptively with changes in tail dispersion—rising automatically when market conditions deteriorate and thereby reducing the likelihood of regulatory intervention or solvency stress due to undercapitalization.

The capital allocation results reveal significant structural differences in tail risk exposures across business units. According to Table 5, all methods assign a relatively higher capital share to risk $X_{2}$, yet STE exhibits stronger sensitivity to tail risk than the traditional ES, especially under heavy-tailed distributions (ν=5) and higher risk-aversion levels (λ=1).

This difference arises not only from the higher mean and variance of $X_{2}$, but more fundamentally from the nonlinear, tail-sensitive covariance structure embedded in the $STE_{p}^{\lambda ,h}$ framework. Specifically, the term $\mathrm{Cov}\left[X_{i},\varpi \left(\frac{F_{S}\left(S\right)-p}{1-p}\right)\right]$ assigns greater weight to extreme tail positions, enhancing the identification of marginal risk contributions under stress conditions. Unlike traditional linear covariance structures, this mechanism captures the structural role of each business unit in systemic risk formation by reflecting their relative positioning within the tail distribution.

From a risk management perspective, this difference translates into substantive allocation adjustments. In the heavy-tailed case (ν=5,λ=1), $EGS_{1.4,p}^{\lambda }$ allocates 36.14% to $X_{2}$, exceeding the 31.50% under $ES_{p}$ by 4.64 percentage points. This gap quantifies $X_{2}$’s marginal contribution to the portfolio’s tail variability: in extreme scenarios, losses on $X_{2}$ are highly positively correlated with the aggregate portfolio, amplifying systemic uncertainty. While ES captures only tail-mean risk, STE further measures the cost of tail variability. This implies that, if capital were allocated solely according to ES, $X_{2}$ could face a capital shortfall in tail-stress conditions: when realized tail losses deviate substantially from their mean, the reserved capital may be insufficient to cover actual losses. By explicitly measuring tail variability, STE provides a capital buffer for such above-mean realizations.

Conversely, for units negatively correlated with the portfolio, the STE approach substantially reduces capital allocations. As shown in Figure 5b,c, under the same parameters (ν,λ)=(5,1), $EGS_{1.4,p}^{\lambda }$ assigns 0.75% and 1.29% to $X_{8}$ and $X_{9}$, respectively, compared with 2.27% and 2.45% under $ES_{p}$, representing decreases of 67% and 47%. This does not underestimate risk. Rather, it quantifies their hedging value in extreme scenarios: tail losses are milder and less synchronized with the portfolio, thereby dampening systemic tail fluctuations. While $ES_{p}$ reflects only mean dependence, STE enhances the identification of structural hedging effects through tail-weighted covariance. This leads to a more reasonable reallocation of capital and avoiding misjudgment of the value of risk-mitigating units that would arise from focusing solely on mean contributions.

As the penalty parameter λ increases from 0.5 to 1.0 (Figure 5a), the allocation to $X_{2}$ under $EGS_{1.4,p}^{\lambda }$ rises from 34.26% to 36.14%, while that to $X_{8}$ decreases from 1.37% to 0.75%. This demonstrates that λ serves as a calibration parameter for tail sensitivity. When tail risk increases, a higher λ directs more capital toward units with greater tail variability. Conversely, when risk subsides or risk aversion decreases, a lower λ makes allocations converge toward $ES_{p}$. This mechanism enables institutions to dynamically adjust capital allocation in response to tail risk conditions and risk preferences.

As the distribution approaches normality (ν=∞), the differences between STE and ES allocations diminish, with $X_{2}$’s allocation under $EGS_{2,p}^{\lambda }$ decreasing from 32.19% (ν=5) to 30.56% (ν=∞). This convergence validates the theoretical consistency of the STE framework: enhanced sensitivity under extreme conditions while maintaining compatibility with traditional methods under normal market environments.

## 7. Conclusions

This paper introduces the shortfall of tail-based entropy (STE), a tail-sensitive risk functional that augments expected shortfall (ES) with a rank-dependent entropy component, thereby capturing tail variability that ES alone may overlook. The framework subsumes several shortfall-type risk measures. We provide equivalent characterizations, derive sufficient conditions for coherence, and establish monotonicity with respect to a tail-variability order. As an application, we characterize the STE-based capital allocation rule and analyze its fundamental properties. We also derive closed-form formulas under a centered-regression representation and relate the rule to the distance-minimization framework of Dhaene et al. [23]. We further obtain explicit expressions for elliptical and extended skew-normal families and discuss several illustrative special cases.

In the empirical analysis, we evaluate the performance of the STE framework for capital allocation using the Panjer [60] insurance dataset. The results indicate that STE offers superior tail identification, higher risk sensitivity, and more accurate recognition of diversification effects compared to traditional methods. The TE component dynamically identifies systemic tail risk exposures across business units. This supports differentiated capital allocations, increasing requirements for high-risk units while appropriately reducing burdens for units that provide hedging effects. The risk-aversion parameter λ offers a flexible adjustment mechanism to represent diverse risk preferences. Under normal market conditions, the allocations generated by STE remain consistent with traditional benchmarks, confirming the framework’s theoretical robustness. Overall, STE constitutes a more precise and effective tool for capital management in environments dominated by tail risk.

We conclude with two directions. First, when raw loss data are available, integrate parameter uncertainty modeling with statistical inference to assess the empirical performance and robustness of STE-based allocations. Second, extend the framework (i) to distributional uncertainty settings with limited information (e.g., known means and covariances) and (ii) to robust risk measurement and capital allocation by leveraging STE’s coherence and dual representation to analyze entropy-induced uncertainty.

## Figures and Tables

**Table 1:** STE risk measures corresponding to specific choices of h∈H.

Name (Notation)	Formula for X∈X and Parameters	Function ${\hat{h}}_{p}^{\lambda }\left(u\right)$
Gini shortfall ($GS_{p}^{\lambda }\left(X\right)$)	$ES_{p}\left(X\right)+\lambda \;TGini_{p}\left(X\right)$λ∈[0,1/2], p∈(0,1)	${\hat{h}}_{p}^{\lambda }\left(u\right)=\frac{{\left(u-p\right)}_{+}}{1-p}+2\lambda (\frac{u-1}{1-p}$ $+{\left(\frac{1-u}{1-p}\right)}^{2})\;I_{\left[p,1\right]}\left(u\right)$
Extended Gini shortfall ($EGS_{r,p}^{\lambda }\left(X\right)$)	$ES_{p}\left(X\right)+\lambda \;TEGini_{r,p}\left(X\right)$$\lambda \in \left[0,\frac{1}{2\left(r-1\right){\left(1-p\right)}^{r-2}}\right]$, r>1, p∈(0,1)	${\hat{h}}_{p}^{\lambda }\left(u\right)=\frac{{\left(u-p\right)}_{+}}{1-p}+2\lambda (\frac{{\left(1-u\right)}^{r}}{{\left(1-p\right)}^{2}}$ $+{\left(1-p\right)}^{r-3}\left(u-1\right))\;I_{\left[p,1\right]}\left(u\right)$
Shortfall of cumulative residual entropy ($CRES_{p}^{\lambda }\left(X\right)$)	$ES_{p}\left(X\right)+\lambda \;TCRE_{p}\left(X\right)$λ∈[0,1], p∈(0,1)	${\hat{h}}_{p}^{\lambda }\left(u\right)=\frac{{\left(u-p\right)}_{+}}{1-p}+\lambda (\frac{1-u}{1-p}\log$ $\frac{1-u}{1-p})\;I_{\left[p,1\right]}\left(u\right)$
Shortfall of right-tail deviation ($RTDS_{p}^{\lambda }\left(X\right)$)	$ES_{p}\left(X\right)+\lambda \;TRTD_{p}\left(X\right)$λ∈[0,2], p∈(0,1)	${\hat{h}}_{p}^{\lambda }\left(u\right)=\frac{{\left(u-p\right)}_{+}}{1-p}-\lambda ({\left(\frac{1-u}{1-p}\right)}^{1/2}$ $-\frac{1-u}{1-p})\;I_{\left[p,1\right]}\left(u\right)$
Shortfall of cumulative residual Tsallis entropy ($CRTES_{\vartheta ,p}^{\lambda }\left(X\right)$)	$ES_{p}\left(X\right)+\lambda \;TCRTE_{\vartheta ,p}\left(X\right)$λ∈[0,1], ϑ>0, ϑ≠1, p∈(0,1)	${\hat{h}}_{p}^{\lambda }\left(u\right)=\frac{{\left(u-p\right)}_{+}}{1-p}-\frac{\lambda }{\vartheta -1}(\frac{1-u}{1-p}$ $-{\left(\frac{1-u}{1-p}\right)}^{\vartheta })\;I_{\left[p,1\right]}\left(u\right)$

**Table 2:** STE-based capital allocation under elliptical distributions.

STE Measure (Notation)	Capital Allocation for $X_{i}$
Gini shortfall ($GS_{p}^{\lambda }\left(X\right)$)	$GS_{p}^{\lambda }\left(X_{i},S\right)=\mu_{i}+\frac{\sigma_{i,S}}{\sigma_{S}}\left[\left(1-2\lambda \right)ES_{p}\left(Z\right)\right.$ $\left.+\frac{4\lambda }{1-p}E\left[\bar{G}\left(Z^{2}/2\right)|Z>z_{p}\right]\right]$
Extended Gini shortfall ($EGS_{R,p}^{\lambda }\left(X\right)$)	$EGS_{R,p}^{\lambda }\left(X_{i},S\right)=\mu_{i}+\frac{\sigma_{i,S}}{\sigma_{S}}\left[\left(1-2\lambda {\left(1-p\right)}^{R-2}\left(R-1\right)\right)\right.$ $\left.\times ES_{p}\left(Z\right)+2\lambda \frac{R(R-1)}{1-p}E\left[F_{Z}{\left(Z\right)}^{R-2}\bar{G}\left(Z^{2}/2\right)|Z>z_{p}\right]\right]$
Shortfall of cumulative residual entropy ($CRES_{p}^{\lambda }\left(X\right)$)	$CRES_{p}^{\lambda }\left(X_{i},S\right)=\mu_{i}+\frac{\sigma_{i,S}}{\sigma_{S}}\left[\left(1-\lambda \right)ES_{p}\left(Z\right)\right.$ $\left.+\lambda E\left[\frac{\bar{G}\left(Z^{2}/2\right)}{F_{Z}\left(Z\right)}|Z>z_{p}\right]\right]$
Shortfall of right-tail deviation ($RTDS_{p}^{\lambda }\left(X\right)$)	$RTDS_{p}^{\lambda }\left(X_{i},S\right)=\mu_{i}+\frac{\sigma_{i,S}}{\sigma_{S}}\left[\left(1-\frac{1}{2}\lambda \right)ES_{p}\left(Z\right)\right.$ $\left.+\frac{1}{4}\lambda {\left(1-p\right)}^{1/2}E\left[{\left(F_{Z}\left(Z\right)\right)}^{-3/2}\bar{G}\left(Z^{2}/2\right)|Z>z_{p}\right]\right]$
Shortfall of cumulative residual Tsallis entropy ($CRTES_{\vartheta ,p}^{\lambda }\left(X\right)$)	$CRTES_{\vartheta ,p}^{\lambda }\left(X_{i},S\right)=\mu_{i}+\frac{\sigma_{i,S}}{\sigma_{S}}\left[\left(1-\lambda \right)ES_{p}\left(Z\right)\right.$ $\left.+\lambda \frac{\vartheta }{{\left(1-p\right)}^{\vartheta -1}}E\left[{\left(F_{Z}\left(Z\right)\right)}^{\vartheta -2}\bar{G}\left(Z^{2}/2\right)|Z>z_{p}\right]\right]$

Note: The corresponding ${\hat{h}}_{p}^{\lambda }\left(u\right)$ functions for each STE measure are provided in Table 1.

**Table 3:** STE-based capital allocation under extended skew-normal distributions.

STE Measure (Notation)	Capital Allocation Formula for $X_{i}$
Gini shortfall ($GS_{p}^{\lambda }\left(X\right)$)	$GS_{p}^{\lambda }\left(X_{i},S\right)=\mu_{i}+\frac{\sigma_{i,S}}{\sigma_{S}}\left[\left(1-2\lambda \right)\;ES_{p}\left(Z\right)+\frac{\lambda }{1-p}\left(4\frac{\alpha_{Z}\eta_{Z}}{c_{Z}}\right.\right.$ $\left.\left.+\;4\;E\left[\left(f_{Z}\left(Z\right)+\frac{\alpha_{Z}\eta_{Z}}{c_{Z}}\;\Phi \left(Zc_{Z}+\frac{\tau_{Z}\alpha_{Z}}{c_{Z}}\right)\right)|Z>z_{p}\right]\right)\right]$ $+\left(1+2\lambda \delta_{i.S}\right)\;E\left[\eta_{i.S}\left(S\right)|S>s_{p}\right]-\frac{4}{1-p}\;\delta_{i.S}\;E\left[\eta_{i.S}\left(S\right)\;{\bar{F}}_{S}\left(S\right)|S>s_{p}\right]$
Extended Gini shortfall ($EGS_{r,p}^{\lambda }\left(X\right)$)	$EGS_{r,p}^{\lambda }\left(X_{i},S\right)=\mu_{i}+\frac{\sigma_{i,S}}{\sigma_{S}}\left(\left(1-2\lambda \left(r-1\right){\left(1-p\right)}^{r-2}\right)ES_{p}\left(Z\right)+\frac{\lambda }{1-p}\left(\frac{2r\alpha_{Z}\eta_{Z}}{c_{Z}}\right.\right.$ $\left.\left.+2r\left(r-1\right)E\left[\left(f_{Z}\left(Z\right)+\frac{\alpha_{Z}\eta_{Z}}{c_{Z}}\Phi \left(Zc_{Z}+\tau_{Z}\alpha_{Z}/c_{Z}\right)\right){\left({\bar{F}}_{Z}\left(Z\right)\right)}^{r-2}|Z>z_{p}\right]\right)\right)$ $+\left(\delta_{i.S}+2\lambda {\left(1-p\right)}^{r-2}\right)E\left[\eta_{i.S}\left(S\right)|S>s_{p}\right]-\frac{2r\lambda }{1-p}\delta_{i.S}E\left[\eta_{i.S}\left(S\right){\left({\bar{F}}_{S}\left(S\right)\right)}^{r-1}|S>s_{p}\right]$
Shortfall of right-tail deviation ($RTDS_{p}^{\lambda }\left(X\right)$)	$RTDS_{p}^{\lambda }\left(X_{i},S\right)=\mu_{i}+\frac{\sigma_{i,S}}{\sigma_{S}}\left(\left(1-\frac{1}{2}\lambda \right)ES_{p}\left(Z\right)-\frac{\lambda }{2(1-p)}\left(\frac{\alpha_{Z}\eta_{Z}}{c_{Z}}-\frac{1}{2}{\left(1-p\right)}^{3/2}\right.\right.$ $\left.\left.\times E\left[\left(f_{Z}\left(Z\right)+\frac{\alpha_{Z}\eta_{Z}}{c_{Z}}\Phi \left(Zc_{Z}+\tau_{Z}\alpha_{Z}/c_{Z}\right)\right){\left({\bar{F}}_{Z}\left(Z\right)\right)}^{-3/2}|Z>z_{p}\right]\right)\right)$ $+\left(\delta_{i.S}-4\lambda \delta_{i.S}\right)\times E\left[\eta_{i.S}\left(S\right)|S>s_{p}\right]+\frac{1}{2}\delta_{i.S}{\left(1-p\right)}^{1/2}E\left[\eta_{i.S}\left(S\right){\bar{F}}_{S}{\left(S\right)}^{-1/2}|S>s_{p}\right]$
Shortfall of cumulative residual Tsallis entropy ($CRTES_{\vartheta ,p}^{\lambda }\left(X\right)$)	$CRTES_{\vartheta ,p}^{\lambda }\left(X_{i},S\right)=\mu_{i}+\frac{\sigma_{i,S}}{\sigma_{S}}\left(\left(1-\lambda \right)ES_{p}\left(Z\right)+\frac{\lambda }{1-p}\left(\frac{\vartheta }{\vartheta -1}\frac{\alpha_{Z}\eta_{Z}}{c_{Z}}+\frac{\vartheta }{{\left(1-p\right)}^{\vartheta -2}}\right.\right.$ $\left.\left.\times E\left[\left(f_{Z}\left(Z\right)+\frac{\alpha_{Z}\eta_{Z}}{c_{Z}}\Phi \left(Zc_{Z}+\tau_{Z}\alpha_{Z}/c_{Z}\right)\right){\left({\bar{F}}_{Z}\left(Z\right)\right)}^{\vartheta -2}|Z>z_{p}\right]\right)\right)$ $+\left(\delta_{i.S}-\lambda \frac{\delta_{i.S}}{\vartheta -1}\right)\times E\left[\eta_{i.S}\left(S\right)|S>s_{p}\right]+\frac{\vartheta \lambda }{\vartheta -1}\frac{\delta_{i.S}}{{\left(1-p\right)}^{\vartheta -1}}E\left[\eta_{i.S}\left(S\right){\bar{F}}_{S}{\left(S\right)}^{\vartheta -1}|S>s_{p}\right]$

Note: The corresponding ${\hat{h}}_{p}^{\lambda }\left(u\right)$ functions for each STE measure are provided in Table 1.

**Table 4:** Comparison of different risk measures under various parameters.

λ	ν	DIV (%)				
		${\mathrm{ES}}_{p}$	${\mathrm{EGS}}_{1.4,p}^{\lambda }$	${\mathrm{EGS}}_{2,p}^{\lambda }$	${\mathrm{CRES}}_{p}^{\lambda }$	${\mathrm{SDS}}_{p}^{\lambda }$
0.5	2	17.01	23.81	21.76	23.75	NaN
	5	11.92	15.17	14.40	14.51	14.53
	*∞*	12.25	12.30	11.82	11.68	11.70
1.0	2	17.01	28.93	25.65	28.69	NaN
	5	11.92	18.04	16.65	16.85	16.89
	*∞*	10.10	14.24	13.43	13.18	13.21

**Table 5:** Capital allocation percentages and aggregate risk based on ES, EGS, and CRES for varying ν, λ, as well as p=0.99.

	λ=0.5							
	ν=5				ν=∞			
	$ES_{p}$	$EGS_{1.4,p}^{\lambda }$	$EGS_{2,p}^{\lambda }$	$CRES_{p}^{\lambda }$	$ES_{p}$	$EGS_{1.4,p}^{\lambda }$	$EGS_{2,p}^{\lambda }$	$CRES_{p}^{\lambda }$
$k_{1}$	17.51%	16.14%	17.34%	17.32%	18.09%	17.62%	18.04%	18.04%
$k_{2}$	31.50%	34.26%	31.85%	31.89%	30.34%	31.29%	30.45%	30.45%
$k_{3}$	0.57%	0.52%	0.56%	0.56%	0.59%	0.57%	0.59%	0.59%
$k_{4}$	9.01%	8.62%	8.96%	8.95%	9.17%	9.04%	9.15%	9.15%
$k_{5}$	0.35%	0.54%	0.37%	0.37%	0.26%	0.33%	0.27%	0.27%
$k_{6}$	18.84%	19.60%	18.94%	18.95%	18.52%	18.78%	18.55%	18.55%
$k_{7}$	10.45%	10.20%	10.42%	10.41%	10.55%	10.47%	10.54%	10.54%
$k_{8}$	2.27%	1.37%	2.15%	2.14%	2.65%	2.34%	2.61%	2.62%
$k_{9}$	2.45%	1.76%	2.36%	2.35%	2.74%	2.50%	2.71%	2.71%
$k_{10}$	7.06%	7.00%	7.05%	7.05%	7.08%	7.06%	7.08%	7.08%
Total	164.084	201.754	168.073	168.504	152.060	161.725	153.147	153.104
	λ=1							
	ν=5				ν=∞			
	$ES_{p}$	$EGS_{1.4,p}^{\lambda }$	$EGS_{2,p}^{\lambda }$	$CRES_{p}^{\lambda }$	$ES_{p}$	$EGS_{1.4,p}^{\lambda }$	$EGS_{2,p}^{\lambda }$	$CRES_{p}^{\lambda }$
$k_{1}$	17.51%	15.20%	17.17%	17.14%	18.09%	18.20%	17.98%	17.99%
$k_{2}$	31.50%	36.14%	32.19%	32.26%	30.34%	32.13%	30.56%	30.55%
$k_{3}$	0.57%	0.48%	0.56%	0.56%	0.59%	0.56%	0.59%	0.59%
$k_{4}$	9.01%	8.36%	8.91%	8.90%	9.17%	8.92%	9.14%	9.14%
$k_{5}$	0.35%	0.67%	0.39%	0.40%	0.26%	0.39%	0.28%	0.28%
$k_{6}$	18.84%	20.12%	19.03%	19.05%	18.52%	19.01%	18.58%	18.58%
$k_{7}$	10.45%	10.03%	10.39%	10.38%	10.55%	10.39%	10.53%	10.53%
$k_{8}$	2.27%	0.75%	2.04%	2.02%	2.65%	2.06%	2.58%	2.58%
$k_{9}$	2.45%	1.29%	2.28%	2.26%	2.74%	2.29%	2.69%	2.69%
$k_{10}$	7.06%	6.96%	7.04%	7.04%	7.08%	7.04%	7.08%	7.08%
Total	164.084	239.424	172.061	172.924	152.060	171.390	154.233	154.148

## Data Availability

Publicly available data were analyzed in this study. The dataset (summary statistics for ten insurance business lines) was originally introduced by Panjer (2002) [60]. We use the published mean vector and covariance matrix reported therein; no new data were generated by the authors. Full parameter values are reproduced in Section 6 of this article.

## Supplementary Materials

The following supporting information can be downloaded at https://www.mdpi.com/article/10.3390/e27111153/s1. References [61,62,63] are cited in the Supplementary Materials.

## Nomenclature

**Symbol**	**Meaning**
${\mathrm{VaR}}_{p}\left(X\right)$, ${\mathrm{ES}}_{p}\left(X\right)$	Value-at-Risk and Expected Shortfall at level *p*
$x_{p}={\mathrm{VaR}}_{p}\left(X\right)$	*p*-quantile of loss *X* (left-continuous inverse)
$X_{p}:=X\;|\;X>x_{p}$	Tail conditional variable beyond $x_{p}$
$F_{X_{p}}$	Distribution function of $X_{p}$
$U_{X}$	Distributional transform of *X* with $F_{X}^{-1}\left(U_{X}\right)=X$ a.s.
h∈H	Concave weighting function with h(0)=h(1)=0
$\varpi =h^{\prime }$	Right derivative of *h*
${\hat{h}}_{p}\left(u\right)$	TE-adjusted weight: 0 for u<p; $-h\;\left((u-p)/(1-p)\right)$ for u≥p
${\hat{h}}_{p}^{\lambda }\left(u\right)$	STE-adjusted weight induced by *h* and λ
λ≥0	Loading parameter in ${\mathrm{STE}}_{p}^{\lambda ,h}$
${\mathrm{TE}}_{p}^{h}\left(X\right)$	Tail-based entropy at level *p*
${\mathrm{STE}}_{p}^{\lambda ,h}\left(X\right)$	Shortfall of tail-based entropy of level *p* and loading λ
$\gamma_{p}^{\lambda }\left(u\right)$	Kernel in the integral representation of ${\mathrm{STE}}_{p}^{\lambda ,h}$

## References

[1] Chakraborty S., Pradhan B. (2023). On weighted cumulative Tsallis residual and past entropy measures. Commun. Stat.-Simul. Comput..

[2] Psarrakos G., Toomaj A., Vliora P. (2024). A family of variability measures based on the cumulative residual entropy and distortion functions. Insur. Math. Econ..

[3] Rajesh G., Sunoj S.M. (2019). Some properties of cumulative Tsallis entropy of order *α*. Stat. Pap..

[4] Calì C., Longobardi M., Navarro J. (2020). Properties for generalized cumulative past measures of information. Probab. Eng. Inform. Sci..

[5] Mohammadi M., Hashempour M. (2024). On weighted version of dynamic cumulative residual inaccuracy measure based on extropy. Stat. Pap..

[6] Zardasht V., Parsi S., Mousazadeh M. (2015). On empirical cumulative residual entropy and a goodness-of-fit test for exponentiality. Stat. Pap..

[7] Haberman S., Khalaf-Allah M., Verrall R. (2011). Entropy, longevity, and the cost of annuities. Insur. Math. Econ..

[8] Yin X., Balakrishnan N., Yin C. (2023). Bounds for Gini’s mean difference based on first four moments, with some applications. Stat. Pap..

[9] Hashempour M., Mohammadi M., Kamari O. (2025). On weighted version of dynamic residual inaccuracy measure using extropy in order statistics with applications in model selection. Stat. Pap..

[10] Toomaj A., Sunoj S.M., Navarro J. (2017). Some properties of the cumulative residual entropy of coherent and mixed systems. J. Appl. Probab..

[11] Furman E., Landsman Z. (2006). Tail variance premium with applications for elliptical portfolio of risks. Astin Bull..

[12] Furman E., Wang R., Zitikis R. (2017). Gini-type measures of risk and variability: Gini shortfall, capital allocations, and heavy-tailed risks. J. Bank Financ..

[13] Hu T., Chen O. (2020). On a family of coherent measures of variability. Insur. Math. Econ..

[14] Berkhouch M., Lakhnati G., Righi M.B. (2018). Extended Gini-type measures of risk and variability. Appl. Math. Financ..

[15] Ben Hssain L., Berkhouch M., Lakhnati G. (2024). Portfolio selection based on extended Gini shortfall risk measures. Stat. Risk Model..

[16] Zuo B., Yin C. (2023). Covariance representations and coherent measures for some entropies. Entropy.

[17] Zuo B., Yin C. (2025). Worst-case distortion riskmetrics and weighted entropy with partial information. Eur. J. Oper. Res..

[18] Tasche D. (1999). Risk Contributions and Performance Measurement.

[19] Tasche D. (2007). Euler Allocation: Theory and Practice. arXiv.

[20] Kim J.H.T., Kim S.Y. (2019). Tail risk measures and risk allocation for the class of multivariate normal mean-variance mixture distributions. Insur. Math. Econ..

[21] Li X., Zhou Y., Zhou Y. (2019). A generalization of expected shortfall based capital allocation. Stat. Probab. Lett..

[22] Marri F., Moutanabbir K. (2022). Risk aggregation and capital allocation using a new generalized Archimedean copula. Insur. Math. Econ..

[23] Dhaene J., Tsanakas A., Valdez E., Vanduffel S. (2012). Optimal capital allocation principles. J. Risk Insur..

[24] Chen E., Wu L., He J. (2024). Dynamic capital allocation with reallocation cost. Oper. Res. Lett..

[25] Boonen T.J. (2020). *τ*-Value for risk capital allocation problems. Oper. Res. Lett..

[26] Yang Y., Wang G., Yao J., Xie H. (2025). A generalized tail mean-variance model for optimal capital allocation. Insur. Math. Econ..

[27] Furman E., Zitikis R. (2008). Weighted risk capital allocations. Insur. Math. Econ..

[28] Ostaszewski K., Xu M. (2012). Optimal Capital Allocation: Mean–Variance Models.

[29] Xu M., Mao T. (2013). Optimal capital allocation based on the tail mean-variance model. Insur. Math. Econ..

[30] Xu M. (2015). TMV-based capital allocation for multivariate risks. Variance.

[31] Bauer D., Zanjani G. (2016). The marginal cost of risk, risk measures, and capital allocation. Manag. Sci..

[32] Balog D., Batyi T.L., Csoka P., Pinter M. (2017). Properties and comparison of risk capital allocation methods. Eur. J. Oper. Res..

[33] Furman E., Kuznetsov A., Zitikis R. (2018). Weighted risk capital allocations in the presence of systematic risk. Insur. Math. Econ..

[34] Boonen T.J., De Waegenaere A., Norde H. (2020). A generalization of the Aumann-Shapley value for risk capital allocation problems. Eur. J. Oper. Res..

[35] Chong W.F., Feng R., Jin L. (2021). Holistic principle for risk aggregation and capital allocation. Ann. Oper. Res..

[36] Grechuk B. (2022). Extended gradient of convex function and capital allocation. Eur. J. Oper. Res..

[37] Jiang Y., Zhao Y., Zhao P. (2023). Optimal capital allocation for individual risk model using a mean-variance principle. J. Ind. Manag. Optim..

[38] Wei L., Hu Y. (2020). Capital allocation with multivariate risk measures: An axiomatic approach. Probab. Eng. Inform..

[39] Wei L., Hu Y. (2023). Capital allocation with multivariate convex risk measures. J. Ind. Manag. Optim..

[40] Guo Q., Bauer D., Zanjani G. (2021). Capital allocation techniques: Review and comparison. Variance.

[41] Mastrogiacomo E., Rosazza Gianin E. (2024). Dynamic capital allocation rules via BSDEs: An axiomatic approach. Ann. Oper. Res..

[42] Li P., Yin C. (2024). The tail mean-variance optimal capital allocation under the extended skew-elliptical distribution. J. Comput. Appl. Math..

[43] Föllmer H., Schied A. (2011). Stochastic Finance: An Introduction in Discrete Time, 3 ed..

[44] Artzner P., Delbaen F., Eber J.M., Heath D. (1999). Coherent measure of risk. Math. Finance.

[45] Wang R., Wei Y., Willmot G.E. (2020). Characterization, robustness, and aggregation of signed Choquet integrals. Math. Oper. Res..

[46] Dhaene J., Goovaerts M. (1996). Dependency of risks and stop-loss order. Astin Bull..

[47] Shaked M., Shanthikumar J. (2007). Stochastic Orders.

[48] Dana R.A. (2005). A representation result for concave Schur-concave functions. Math. Financ..

[49] Cherny A. (2009). Capital allocation and risk contribution with discrete-time coherent risk. Math. Financ..

[50] Cherny A., Orlov D. (2011). On two approaches to coherent risk contribution. Math. Financ..

[51] Tsanakas A. (2008). Risk measurement in the presence of background risk. Insur. Math. Econ..

[52] Cai J., Wang Y. (2021). Optimal capital allocation principles considering capital shortfall and surplus risks in a hierarchical corporate structure. Insur. Math. Econ..

[53] Wang W., Xu H., Ma T. (2023). Optimal scenario-dependent multivariate shortfall risk measure and its application in risk capital allocation. Eur. J. Oper. Res..

[54] Sandström A. (2010). Handbook of Solvency for Actuaries and Risk Managers: Theory and Practice.

[55] Cannata F., Quagliariello M. (2011). Basel III and Beyond.

[56] Rüschendorf L. (2013). Mathematical Risk Analysis. Dependence, Risk Bounds, Optimal Allocations and Portfolios.

[57] Tsanakas A., Millossovich P. (2016). Sensitivity analysis using risk measures. Risk Anal..

[58] McNeil A.J., Frey R., Embrechts P. (2015). Quantitative Risk Management: Concepts, Techniques and Tools.

[59] Capitanio A., Azzalini A., Stanghellini E. (2003). Graphical models for skew-normal variates. Scand. J. Stat..

[60] Panjer H. (2002). Measurement of Risk, Solvency Requirements, and Allocation of Capital Within Financial Conglomerates.

[61] Tsanakas A. (2004). Dynamic capital allocation with distortion risk measures. Insur. Math. Econ..

[62] Landsman Z., Valdez E. (2003). Tail conditional expectations for elliptical distributions. N. Am. Actuar. J..

[63] Fang K.T., Kotz S., Ng K.W. (1990). Symmetric Multivariate and Related Distributions.

