# Supplementary material for: On the Shortfall of Tail-Based Entropy and Its Application to Capital Allocation
Source: Entropy (Basel). 2025 Nov 13;27(11):1153. doi: 10.3390/e27111153 (PMC12651032; doi:10.3390/e27111153)
Supplement: Supplementary file 1 [file entropy-27-01153-s001.zip › entropy-3967985-supplementary.pdf]

Supplementary Material for "On the Shortfall of Tail-Based Entropy and Its Application to Capital Allocation"

Pingyun Li and Chuancun Yin *
School of Statistics and Data Science, Qufu Normal University,
Qufu 273165, China

*Corresponding author. E-mail: ccyin@qfnu.edu.cn;
Contributing author: pingyun31@gmail.com

entropy - Supplementary Material

This Supplementary Material provides detailed proofs and technical lemmas that support the theoretical developments of the paper. The material is organized to correspond with the main results presented in the text.

S.1 Proofs for Section 3: Shortfall of tail-based framework

S.1.1 Proof of Theorem 1

Proof Write $X_p = F_X^{-1}(U_p)$ and $Y_p = F_Y^{-1}(U_p)$, where U_p is uniformly distributed on $[p,1]$. Since the IE is CX-monotone (main paper, Remark 3) and $X_p \preceq_{\mathrm{CX}} Y_p$, together with $\mathrm{TE}_p^h(X) = \mathrm{IE}^h(X_p)$, we have $\mathrm{IE}^h(X_p) \leq \mathrm{IE}^h(Y_p)$; thus $\mathrm{TE}_p^h(X) \leq \mathrm{TE}_p^h(Y)$. □

S.1.2 Proof of Theorem 2

Proof (1) From the signed Choquet integral representation we have

$$\mathrm{STE}_p^{\lambda,h}(X) = \int_0^1 F_X^{-1}(u)\, d\hat{h}_p^{\lambda}(u) = \int_0^1 F_X^{-1}(u)\, \gamma_p^{\lambda}(u)\, du.$$

Let $U_X \sim U[0,1]$ with $F_X^{-1}(U_X) = X$ almost surely, this yields the equivalent expectation form $\mathrm{E}\big[X\gamma_p^{\lambda}(U_X)\big]$.

(2) Property (2) follows directly from the properties of signed Choquet integrals (main paper, Remark 2).

(3) Noting that $\gamma_p^{\lambda}(u) = 0$ for $u \in [0, p)$, and that since h is concave on $[0, 1]$, $\gamma_p^{\lambda}(u)$ is an increasing function on $[p, 1]$, elementary analysis shows that $\gamma_p^{\lambda}(u)$ is non-negative if and only if $\lambda \in \left[0, \frac{1}{\varpi(0)}\right]$, $\varpi(0) \neq 0$. By Lemma 4.2 of Furman et al. [1], we conclude that $\mathrm{STE}_p^{\lambda,h}$ is monotone if and only if $\gamma_p^{\lambda}(u)$ is non-negative for all $u \in [0, 1]$. Additionally, STE_p^{λ} is subadditive if and only if $\gamma_p^{\lambda}(u)$ is non-decreasing for $u \in [0, 1]$. Therefore, we establish the following equivalences: *(i)* $\Leftrightarrow$ *(ii)* $\Leftrightarrow$ *(v)*. The equivalence *(iv)* $\Leftrightarrow$ *(i)* + *(ii)* is trivial because STE_p^{λ} satisfies translation invariance and positive homogeneous. It is known from Corollary 4.65 of Föllmer and Schied [2] that a law-invariant coherent risk measure preserves the increasing convex order. This reveals *(iv)* $\Leftrightarrow$ *(iii)*. This proves that all statements *(i)*$-$*(v)* are equivalent, and thus completes the proof of Theorem 2. □

S.2 Proofs for Section 4: The STE-based allocation

S.2.1 Auxiliary lemma for Proposition 3

Lemma S.2.1. *(Tsanakas [3]). Let (X_1, X_2) and (Y_1, Y_2) be two random vectors such that $X_1 \overset{d}{=} Y_1$ and $X_2 \overset{d}{=} Y_2$. If the (Y_1, Y_2) is comonotonic, then $(X_1, X_2) \preceq_{\mathrm{corr}} (Y_1, Y_2)$ and for any non-decreasing functions h_1, h_2 for which the expectations exist, the following inequality holds:*

$$E[h_1(X_1)h_2(X_2)] \leq E[h_1(Y_1)h_2(Y_2)].$$

S.2.2 Proof of Proposition 3

Proof (1) Using $\mathrm{E}[\gamma_p^{\lambda}(U_S)] = 1$ and

$$\mathrm{STE}_p^{\lambda,h}(X_i, S) = \mathrm{E}\big[X_i\, \gamma_p^{\lambda}(U_S)\big] = \mathrm{Cov}[X_i, \gamma_p^{\lambda}(U_S)] + \mathrm{E}[X_i],$$

the result follows.

(2) Since $\boldsymbol{X} = (X_1, \ldots, X_n)$ is comonotonic, there exists a uniform random variable $U \sim U[0, 1]$ such that

$$X_i = F_{X_i}^{-1}(U), \quad i = 1, \ldots, n, \qquad S = F_S^{-1}(U),$$

see Dhaene et al. [4]. In particular, each pair (X_i, S) is comonotonic. With continuous marginals this is equivalent to $F_{X_i}(X_i) = F_S(S) = U$. Let $U_S = F_S(S)$. Then, by the definition of the allocation,

$$\begin{aligned}
\mathrm{STE}_p^{\lambda,h}(X_i, S) &= \mathrm{E}\left[X_i\, \gamma_p^{\lambda}(U_S)\right] \\
&= \mathrm{E}\left[X_i\, \gamma_p^{\lambda}(F_S(S))\right] \\
&= \mathrm{E}\Big[X_i\, \gamma_p^{\lambda}(F_{X_i}(X_i))\Big] = \mathrm{STE}_p^{\lambda,h}(X_i).
\end{aligned}$$

(3) Let U_S and U_{X_i} denote distributional transform variables of S and X_i (so $U_S, U_{X_i} \sim U[0, 1]$ and $F_S^{-1}(U_S) = S$, $F_{X_i}^{-1}(U_{X_i}) = X_i$ almost surely).

When $\lambda \in [0, 1/\varpi(0)]$ with $\varpi(0) \neq 0$, Theorem 2 implies that γ_p^{λ} is non-decreasing. Consider the two pairs $(X_i, \gamma_p^{\lambda}(U_{X_i}))$ and $(X_i, \gamma_p^{\lambda}(U_S))$. Since $(X_i, \gamma_p^{\lambda}(U_{X_i}))$ is comonotonic and $\gamma_p^{\lambda}(U_{X_i}) \overset{d}{=} \gamma_p^{\lambda}(U_S)$, we have

$$(X_i, \gamma_p^{\lambda}(U_S)) \;\preceq_{\mathrm{corr}}\; (X_i, \gamma_p^{\lambda}(U_{X_i})).$$

Lemma S.2.1 implies

$$\mathrm{STE}_p^{\lambda,h}(X_i,S)=\mathrm{E}[X_i\gamma_p^{\lambda}(U_S)]\le \mathrm{E}[X_i\gamma_p^{\lambda}(U_{X_i})]=\mathrm{STE}_p^{\lambda,h}(X_i).$$

(4) The conclusion follows directly by applying Lemma S.2.1 as in (3). □

S.2.3 Proof of Theorem 4

Proof The first component, $\mathrm{ES}_p(X_i,S)$, is the conditional expectation $\mathrm{E}[X_i|U_S>p]$. By the law of total expectation and using the centered regression function assumption, we have

$$\begin{aligned}\mathrm{ES}_p(X_i,S)&=\mathrm{E}[\mathrm{E}[X_i|S]|U_S>p]\\&=\mathrm{E}[\mathrm{E}[X_i]+C(F_{X_i,S})(S-\mathrm{E}[S])|U_S>p]\\&=\mathrm{E}[X_i]+C(F_{X_i,S})(\mathrm{ES}_p(S)-\mathrm{E}[S]).\end{aligned}\tag{S.2.1}$$

The second component, $\mathrm{TE}_p^h(X_i,S)$, is defined as $-\mathrm{Cov}[X_i,\varpi(\frac{U_S-p}{1-p})|U_S>p]$. Let $\varpi_S=\varpi\left(\frac{U_S-p}{1-p}\right)$ for notational simplicity. Using the property of covariance and again applying the linear regression assumption, we get

$$\begin{aligned}\mathrm{TE}_p^h(X_i,S)&=\mathrm{E}[(\mathrm{ES}_p(X_i,S)-X_i)\varpi_S|U_S>p]\\&=\mathrm{E}[(\mathrm{ES}_p(X_i,S)-\mathrm{E}[X_i])\varpi_S|U_S>p]-\mathrm{E}[(X_i-\mathrm{E}[X_i])\varpi_S|U_S>p].\end{aligned}\tag{S.2.2}$$

Direct calculations give

$$\begin{aligned}\mathrm{E}[(\mathrm{ES}_p(X_i,S)-\mathrm{E}[X_i])\varpi_S|U_S>p]&=(\mathrm{ES}_p(X_i,S)-\mathrm{E}[X_i])\int_p^1\frac{1}{1-p}\varpi\left(\frac{u-p}{1-p}\right)du\\&=(\mathrm{ES}_p(X_i,S)-\mathrm{E}[X_i])\,(h(1)-h(0))\\&=0.\end{aligned}$$

Eq. (S.2.2) implies

$$\begin{aligned}\mathrm{TE}_p^h(X_i,S)&=-\mathrm{E}[(X_i-\mathrm{E}[X_i])\varpi_S|U_S>p]\\&=-\mathrm{E}[\mathrm{E}[X_i-\mathrm{E}[X_i]|S]\varpi_S|U_S>p]\\&=-C(F_{X_i,S})\mathrm{E}[(S-\mathrm{E}[S])\varpi_S|U_S>p]\\&=C(F_{X_i,S})\left(\mathrm{E}\left[(\mathrm{E}[S]-\mathrm{ES}_p(S))\,\varpi_S\middle|U_S>p\right]-\mathrm{E}\left[(S-\mathrm{ES}_p(S))\varpi_S\middle|U_S>p\right]\right)\\&=C(F_{X_i,S})\left(-\mathrm{Cov}\left[S,\varpi_S|U_S>p\right]\right)\\&=C(F_{X_i,S})\,\mathrm{TE}_p^h(S).\end{aligned}\tag{S.2.3}$$

Finally, substituting (S.2.1) and (S.2.3) back into the definition of STE-based allocation

$$\mathrm{STE}_p^{\lambda,h}(X_i,S)=\mathrm{E}[X_i]+C(F_{X_i,S})\left(\mathrm{STE}_p^{\lambda,h}(S)-\mathrm{E}[S]\right).\tag{S.2.4}$$

This completes the proof. □

S.2.4 Proof of Proposition 5

Proof For $D(x)=x^2$, the optimal allocation takes the following form (Dhaene et al. [4])

$$K_i=\mathrm{E}[\zeta_iX_i]+v_i\left(K-\sum_{j=1}^n\mathrm{E}[\zeta_jX_j]\right),\quad i=1,\ldots,n.$$

Substituting $\zeta_i = \gamma_p^{\lambda}(U_S)$ and $v_i = \dfrac{\mathrm{E}[\zeta X_i]}{\sum_{j=1}^n \mathrm{E}[\zeta X_j]}$, we obtain

$$K_i = \mathrm{E}[\zeta X_i] + \frac{\mathrm{E}[\zeta X_i]}{\sum_{j=1}^n \mathrm{E}[\zeta X_j]}\left(K - \sum_{j=1}^{n} \mathrm{E}[\zeta X_j]\right) = \frac{\mathrm{E}[\zeta X_i]}{\sum_{j=1}^n \mathrm{E}[\zeta X_j]} K.$$

For $\lambda \in \left[0, \frac{1}{\varpi(0)}\right]$, Theorem 2 ensures $\gamma_p^{\lambda}(u) \geq 0$, hence $\zeta_i \geq 0$ as required. Since $\mathrm{E}[\zeta X_i] = \mathrm{STE}_p^{\lambda,h}(X_i, S)$ and $K = \sum_{j=1}^n \mathrm{STE}_p^{\lambda,h}(X_j, S)$, we have

$$K_i = \frac{\mathrm{STE}_p^{\lambda,h}(X_i, S)}{\sum_{j=1}^n \mathrm{STE}_p^{\lambda,h}(X_j, S)} K = \mathrm{STE}_p^{\lambda,h}(X_i, S).$$

Hence, the optimal allocation coincides with the STE-based allocation. □

S.3 Proofs for Section 5: STE-based allocation for some parametric distribution

S.3.1 Auxiliary lemmas for Theorem 6

Lemma S.3.1. *(Landsman and Valdez [5]) Let $\boldsymbol{X} \sim \mathrm{E}_n(\boldsymbol{\mu}, \boldsymbol{\Sigma}, g_n)$. Then for $1 \leq i \leq n$, the vector $\boldsymbol{X}_{i,S} = (X_i, S)^T$ has an elliptical distribution with the same generator, that is, $\boldsymbol{X}_{i,S} \sim \mathrm{E}_2(\boldsymbol{\mu}_{i,S}, \boldsymbol{\Sigma}_{i,S}, g_2)$, where $\boldsymbol{\mu}_{i,S} = (\mu_i, \sum_{i=1}^n \mu_j)^T$,*

$$\boldsymbol{\Sigma}_{i,S} = \begin{pmatrix} \sigma_i^2 & \sigma_{i,S} \\ \sigma_{i,S} & \sigma_S^2 \end{pmatrix},$$

and $\sigma_i^2 = \sigma_{ii}$, $\sigma_{i,S} = \sum_{j=1}^n \sigma_{ij}$, $\sigma_S^2 = \sum_{i,j=1}^n \sigma_{ij}$.

Lemma S.3.2. *Consider $Z \sim S(g_1)$ with finite mean and variance, under the assumptions that $h \in \mathcal{H}$ is twice differentiable, and $\varpi(0)$ exists. Then for every $p \in (0,1)$, we have*

$$\mathrm{ES}_p(Z) = \frac{\overline{G}(z_p^2/2)}{1-p}, \tag{S.3.1}$$

and

$$\mathrm{TE}_p^h(Z) = -\varpi(0)\mathrm{ES}_p(Z) - \frac{1}{1-p}\mathrm{E}\left[\varpi'\left(\frac{F_Z(Z)-p}{1-p}\right)\overline{G}(Z^2/2)\middle| Z > z_p\right]. \tag{S.3.2}$$

Proof Eq. (S.3.1) can easily be established by using the definition of the pdf of Z and then appropriately changing the variable of integration. Next, we need to verify formula (S.3.2)

$$\begin{aligned}\mathrm{TE}_p^h(Z) &= -\operatorname{Cov}\left[Z, \varpi\left(\frac{F_Z(Z)-p}{1-p}\right)|Z > z_p\right] \\ &= -\mathrm{E}\left[Z\varpi\left(\frac{F_Z(Z)-p}{1-p}\right)|Z > z_p\right]\end{aligned}$$

$$= -\frac{1}{1-p}\int_{z_p}^{\infty} z\varpi\left(\frac{F_Z(z)-p}{1-p}\right) f(z)\,dz.$$

Next, we use $zf(z)\,dz = -\,d\overline{G}(z^2/2)$, integration by parts, we arrive at

$$\begin{aligned}\mathrm{TE}_p^h(Z) =& \frac{1}{1-p}\int_{z_p}^{\infty} \varpi\left(\frac{F_Z(z)-p}{1-p}\right)\, d\overline{G}(z^2/2)\\ =& -\frac{1}{1-p}\varpi(0)\overline{G}(z_p^2/2) - \frac{1}{(1-p)^2}\int_{z_p}^{\infty} f(z)\varpi'\left(\frac{F_Z(z)-p}{1-p}\right)\overline{G}(z^2/2)\,dz\\ =& -\varpi(0)\mathrm{ES}_p(Z) - \frac{1}{1-p}\mathrm{E}\left[\varpi'\left(\frac{F_Z(Z)-p}{1-p}\right)\overline{G}(Z^2/2)\big|Z > z_p\right].\end{aligned}$$

This completes the proof. □

Lemma S.3.3. *Consider $\boldsymbol{X} \sim \mathrm{E}_n(\boldsymbol{\mu}, \boldsymbol{\Sigma}, g_n)$ with finite mean and covariance, under the assumptions that $h \in \mathcal{H}$ is twice differentiable, and $\varpi(0)$ exists. Then for every $p \in (0,1)$, we have*

$$\mathrm{ES}_p(S) = \mu_S + \frac{\overline{G}(z_p^2/2)}{1-p}\sigma_S, \tag{S.3.3}$$

and

$$\mathrm{TE}_p^h(S) = -\sigma_S\left[\varpi(0)\mathrm{ES}_p(Z) + \frac{1}{1-p}\mathrm{E}\left[\varpi'\left(\frac{F_Z(Z)-p}{1-p}\right)\overline{G}(Z^2/2)\big|Z > z_p\right]\right]. \tag{S.3.4}$$

Proof Recall (e.g. Fang et al. [6]) that $S \sim \mathrm{E}_1(\mu_S, \sigma_S^2, g)$. Hence, Eqs. (S.3.3) and (S.3.4) follow from Lemma S.3.2 as follows

$$\mathrm{ES}_p(S) = \mathrm{ES}_p(\mu_S + \sigma_S Z) = \mu_S + \sigma_S\mathrm{ES}_p(Z),$$

and

$$\mathrm{TE}_p^h(S) = \mathrm{TE}_p^h(\mu_S + \sigma_S Z) = \sigma_S\mathrm{TE}_p^h(Z).$$

This completes the proof. □

S.3.2 Proof of Theorem 6

Proof From Dhaene et al. [4], the centered regression function $\gamma_{X_i|S}(s)$ is linear, which is given by

$$\gamma_{X_i|S}(s) = \frac{\sigma_{i,S}}{\sigma_S^2}(s-\mu_S),$$

then we have representation $\gamma_{X_i|S} = C(F_{X_i,S})(s - \mathrm{E}[S])$ with

$$C(F_{X_i,S}) = \frac{\sigma_{i,S}}{\sigma_S^2}. \tag{S.3.5}$$

Hence, from Theorem 4 and Lemma S.3.3, we complete the proof. □

S.3.3 Proof of Lemma 1

Proof Let $\boldsymbol{X} \sim \mathrm{ESN}_n(\boldsymbol{\mu}, \boldsymbol{\Sigma}, \tau, \boldsymbol{\alpha})$. Then

$$\boldsymbol{Y} = B_i \boldsymbol{X} = (X_i, S)^T \sim \mathrm{ESN}_2(\boldsymbol{\mu}_{i,S}, \boldsymbol{\Sigma}_{i,S}, \tau_{i,S}, \boldsymbol{\alpha}_{i,S}),$$

where

$$B_i = \begin{pmatrix} 0 \dots 1 \dots 0 \dots 0 \\ 1 \dots 1 \dots 1 \dots 1 \end{pmatrix},$$

which consists of zeros in the first row except the i-th column with value 1, and all ones in the second row, and

$$\begin{aligned}
\boldsymbol{\mu}_{i,S} &= B_i \boldsymbol{\mu} = (\mu_i, \mu_S)^T, \qquad \mu_S = \sum_{i=1}^{n} \mu_i, \\
\boldsymbol{\Sigma}_{i,S} &= B_i \boldsymbol{\Sigma} B_i^T = \begin{pmatrix} \sigma_i^2 & \sigma_{i,S} \\ \sigma_{i,S} & \sigma_S^2 \end{pmatrix}, \\
\tau_{i,S} &= \frac{\tau}{\left[1 + \boldsymbol{\alpha}^T \left(\boldsymbol{\Sigma} - \boldsymbol{\Sigma} B_i^T \boldsymbol{\Sigma}_{i,S}^{-1} B_i \boldsymbol{\Sigma}\right) \boldsymbol{\alpha}\right]^{1/2}}, \\
\boldsymbol{\alpha}_{i,S} &= \frac{\boldsymbol{\Sigma}_{i,S}^{-1} B_i \boldsymbol{\Sigma} \boldsymbol{\alpha}}{\left[1 + \boldsymbol{\alpha}^T \left(\boldsymbol{\Sigma} - \Sigma B_i^T \boldsymbol{\Sigma}_{i,S}^{-1} B_i \boldsymbol{\Sigma}\right) \boldsymbol{\alpha}\right]^{1/2}} = (\alpha_i^*, \alpha_S^*)^T.
\end{aligned}$$

By substituting the density generator of the extended skew-elliptical distribution with that of the extended skew-normal distribution, as established in Lemma 9 of Li and Yin [7], we prove Lemma 1. □

S.3.4 Auxiliary lemma for Theorem 7

Lemma S.3.4. *Consider* $\boldsymbol{X} \sim \mathrm{ESN}_n(\boldsymbol{\mu}, \boldsymbol{\Sigma}, \tau, \boldsymbol{\alpha})$ *with finite mean and covariance, under the assumptions that* $h \in \mathcal{H}$ *is twice differentiable, and* $\varpi(0)$, $\varpi(1)$ *both exist. Then for every* $p \in (0, 1)$, *we have*

$$\mathrm{ES}_p(S) = \frac{\sigma_S}{1-p} \left(f_Z(z_p) + \frac{\alpha_Z \eta_Z}{c_Z} \overline{\Phi} \left(c_Z z_p + \frac{\tau_Z \alpha_Z}{c_Z} \right) \right) + \mu_S, \tag{S.3.6}$$

and

$$\begin{aligned}
\mathrm{TE}_p^h(S) = &- \sigma_S \left(\frac{1}{1-p} \left(\mathrm{E} \left[\left(f_Z(Z) + \frac{\alpha_Z \eta_Z}{c_Z} \Phi(Z c_Z + \tau_Z \alpha_Z / c_Z) \right) \varpi' \left(\frac{F_Z(Z) - p}{1-p} \right) | Z > z_p \right] \right.\right. \\
&\left.\left. + \frac{\alpha_Z \eta_Z}{c_Z} \left(\varpi(1) - \varpi(0) \right) \right) + \varpi(0) \mathrm{ES}_p(Z) \right). \qquad \text{(S.3.7)}
\end{aligned}$$

Proof In particular, with $\boldsymbol{e} = (1, \dots, 1)^\top$ set $S = \boldsymbol{e}^\top \boldsymbol{X}$ and $Z = (S - \mu_S)/\sigma_S$. Consequently, $S \sim \mathrm{ESN}_1(\mu_S, \sigma_S^2, \tau_S, \alpha_S)$ and $Z \sim \mathrm{ESN}_1(0, 1, \tau_Z, \alpha_Z)$, where $\mu_S = \boldsymbol{e}^\top \boldsymbol{\mu}$, $\sigma_S^2 = \boldsymbol{e}^\top \boldsymbol{\Sigma} \boldsymbol{e}$, $\kappa_S = \left[1 + \boldsymbol{\alpha}^\top (\boldsymbol{\Sigma} - \sigma_S^{-2} \boldsymbol{\Sigma} \boldsymbol{e} \boldsymbol{e}^\top \boldsymbol{\Sigma}) \boldsymbol{\alpha}\right]^{1/2}$, $\tau_Z = \kappa_S^{-1} \tau$, $\alpha_Z = \kappa_S^{-1} \sigma_S^{-1} \boldsymbol{e}^\top \boldsymbol{\Sigma} \boldsymbol{\alpha}$ (so that $\tau_S = \tau_Z$ and $\alpha_S = \sigma_S^{-1} \alpha_Z$). Then

$$\mathrm{ES}_p(Z) = \frac{1}{1-p} \int_{z_p}^{\infty} z f_Z(z) \, dz$$

$$
\begin{aligned}
&= \frac{1}{1-p} \frac{1}{\Phi(\tau_Z/c_Z)} \int_{z_p}^{\infty} z\phi(z)\Phi(\tau_Z + \alpha_Z z)\, dz \\
&= \frac{1}{1-p} \frac{1}{\Phi(\tau_Z/c_Z)} \left[\Phi(\tau_Z + \alpha_Z z_p)\phi(z_p) + \alpha_Z \int_{z_p}^{\infty} \phi(\tau_Z + \alpha_Z z)\phi(z)\, dz \right] \\
&= \frac{1}{1-p} \left(f_Z(z_p) + \frac{\alpha_Z \eta_Z}{c_Z} \overline{\Phi} \left(c_Z z_p + \frac{\tau_Z \alpha_Z}{c_Z} \right) \right),
\end{aligned} \tag{S.3.8}
$$

where $c_Z = (1+\alpha_Z^2)^{1/2}$ and $\eta_Z = \frac{\phi(\tau_Z/c_Z)}{\Phi(\tau_Z/c_Z)}$.

$$
\begin{aligned}
\mathrm{TE}_p^h(Z) =& - \mathrm{Cov}\left[Z, \varpi\left(\frac{F_Z(Z)-p}{1-p} \right) |Z > z_p \right] \\
=& - \mathrm{E}\left[Z\varpi\left(\frac{F_Z(Z)-p}{1-p} \right) |Z > z_p \right] \\
=& - \frac{1}{1-p} \int_{z_p}^{\infty} z\varpi\left(\frac{F_Z(z)-p}{1-p} \right) f_Z(z)\, dz \\
=& - \frac{1}{1-p} \frac{1}{\Phi(\tau_Z/c_Z)} \int_{z_p}^{\infty} z\phi(z)\Phi(\tau_Z + \alpha_Z z)\varpi\left(\frac{F_Z(z)-p}{1-p} \right) dz \\
=& - \frac{\varpi(0)}{1-p} f_Z(z_p) - \frac{1}{1-p} \frac{1}{\Phi(\tau_Z/c_Z)} \left(\alpha_Z \int_{z_p}^{\infty} \phi(\tau_Z + \alpha_Z z)\phi(z)\varpi\left(\frac{F_Z(z)-p}{1-p} \right) dz \right. \\
& \left. + \frac{1}{1-p} \int_{z_p}^{\infty} \Phi(\tau_Z + \alpha_Z z)\phi(z)\varpi'\left(\frac{F_Z(z)-p}{1-p} \right) f_Z(z)\, dz \right) \\
=& - \frac{\varpi(0)}{1-p} f_Z(z_p) - \frac{1}{1-p} \frac{\alpha_Z \eta_Z}{c_Z} \left(\varpi(1) - \varpi(0)\Phi\left(c_Z z_p + \frac{\tau_Z \alpha_Z}{c_Z} \right) \right. \\
& \left. + \mathrm{E}\left[\Phi\left(Z c_Z + \frac{\tau_Z \alpha_Z}{c_Z} \right) \varpi'\left(\frac{F_Z(Z)-p}{1-p} \right) |Z > z_p \right] \right) \\
& - \frac{1}{1-p} \mathrm{E}\left[f_Z(Z)\varpi'\left(\frac{F_Z(Z)-p}{1-p} \right) |Z > z_p \right] \\
=& - \frac{1}{1-p} \left[\mathrm{E}\left[\left(f_Z(Z) + \frac{\alpha_Z \eta_Z}{c_Z} \Phi(Z c_Z + \tau_Z \alpha_Z / c_Z) \right) \varpi'\left(\frac{F_Z(Z)-p}{1-p} \right) |Z > z_p \right] \right. \\
& \left. + \frac{\alpha_Z \eta_Z}{c_Z} (\varpi(1) - \varpi(0)) \right] - \varpi(0)\mathrm{ES}_p(Z).
\end{aligned} \tag{S.3.9}
$$

Note

$$
\mathrm{TE}_p^h(S) = \mathrm{TE}_p^h(\sigma_S Z + \mu_S).
$$

Hence, Eqs (S.3.6) and (S.3.7) follow from Eqs (S.3.8) and (S.3.9) as follows

$$
\mathrm{ES}_p(S) = \mathrm{ES}_p(\mu_S + \sigma_S Z) = \mu_S + \sigma_S \mathrm{ES}_p(Z),
$$

and

$$
\mathrm{TE}_p^h(S) = \mathrm{TE}_p^h(\mu_S + \sigma_S Z) = \sigma_S \mathrm{TE}_p^h(Z).
$$

This completes the proof. □

S.3.5 Proof of Theorem 7

Proof The first component, $\mathrm{ES}_p(X_i, S)$, is the conditional expectation $\mathrm{E}[X_i|S > s_p]$. From the law of total probability we find that

$$
\mathrm{ES}_p(X_i, S) = \int_{s_p}^{\infty} \mathrm{E}[X_i|S = s] dF(s|S > s_p). \tag{S.3.10}
$$

Substituting the expression $\mathrm{E}[X_i|S=s] = s\frac{\sigma_{i,S}}{\sigma_S^2} + \delta_{i.S}\eta_{i.S}(s) + k_i$ (Lemma 1) in (S.3.10) leads to

$$\mathrm{ES}_p(X_i, S) = \mu_i + \frac{\sigma_{i,S}}{\sigma_S}\mathrm{ES}_p(Z) + \delta_{i.S}\mathrm{E}\left[\eta_{i.S}(S)|S > s_p\right]. \tag{S.3.11}$$

The second component, $\mathrm{TE}_p^h(X_i, S)$, is defined as $-\mathrm{Cov}[X_i, \varpi(\frac{F_S(S)-p}{1-p})|S > s_p]$. Recall that

$$X_i = \boldsymbol{e}_i^T \boldsymbol{X} \sim \mathrm{ESN}_1(\mu_i, \sigma_i^2, \tau_i, \alpha_i),$$

where $\boldsymbol{e}_i = (0\ldots1\ldots0)^T \in \mathbb{R}^n$, which consists of zeros except the i-th column with value 1, and $\tau_i = \frac{\tau}{[1+\boldsymbol{\alpha}^T(\boldsymbol{\Sigma}-\sigma_i^{-2}\boldsymbol{\Sigma}\boldsymbol{e}_i\boldsymbol{e}_i^T\boldsymbol{\Sigma})\boldsymbol{\alpha}]^{1/2}}$, $\alpha_i = \frac{\sigma_{ii.S}^{-1}\boldsymbol{e}_i^T\boldsymbol{\Sigma}}{[1+\boldsymbol{\alpha}^T(\boldsymbol{\Sigma}-\sigma_i^{-2}\boldsymbol{\Sigma}\boldsymbol{e}_i\boldsymbol{e}_i^T\boldsymbol{\Sigma})\boldsymbol{\alpha}]^{1/2}}\boldsymbol{\alpha}$. Hence

$$\mathrm{E}[X_i] = \mu_i + \eta_i\delta_i,$$

where $\delta_i = c_i^{-1}\sigma_i^2\alpha_i$ and $\eta_i = \frac{\phi(\tau_i/c_i)}{\Phi(\tau_i/c_i)}$ with $c_i = \left(1 + (\alpha_i\sigma_i)^2\right)^{1/2}$. Further,

$$\begin{aligned}
\mathrm{TE}_p^h(X_i, S) =& -\mathrm{Cov}\left[X_i, \varpi\left(\frac{F_S(S)-p}{1-p}\right)|S > s_p\right] \\
=& -\mathrm{E}\left[(X_i - \mathrm{E}[X_i])\,\varpi\left(\frac{F_S(S)-p}{1-p}\right)|S > s_p\right] \\
=& -\mathrm{E}\left[\left(S\frac{\sigma_{i,S}}{\sigma_S^2} + \delta_{i.S}\eta_{i.S}(S)\right)\varpi\left(\frac{F_S(S)-p}{1-p}\right)|S > s_p\right] \\
&+ \left(\mu_S\frac{\sigma_{i,S}}{\sigma_S^2} + \eta_i\delta_i\right)\mathrm{E}\left[\varpi\left(\frac{F_S(S)-p}{1-p}\right)|S > s_p\right] \\
=& \frac{\sigma_{i,S}}{\sigma_S}\mathrm{TE}_p^h(Z) - \delta_{i.S}\mathrm{E}\left[\eta_{i.S}(S)\varpi\left(\frac{F_S(S)-p}{1-p}\right)|S > s_p\right].
\end{aligned} \tag{S.3.12}$$

Finally, substituting (S.3.11) and (S.3.12) back into the definition of STE-based allocation yileds

$$\begin{aligned}
\mathrm{STE}_p^{\lambda,h}(X_i, S) =& \mu_i + \delta_{i.S}\mathrm{E}\left[\eta_{i.S}(S)\left(1 - \lambda\,\varpi\left(\frac{F_S(S)-p}{1-p}\right)\right)|S > s_p\right] \\
&+ \frac{\sigma_{i,S}}{\sigma_S}\left(\mathrm{ES}_p(Z) + \lambda\,\mathrm{TE}_p^h(Z)\right).
\end{aligned}$$

This completes the proof. □

References

[1] Furman, E., Wang, R., Zitikis, R.: Gini-type measures of risk and variability: Gini shortfall, capital allocations, and heavy-tailed risks. J. Bank Financ. **83**, 70–84 (2017) https://doi.org/10.1016/j.jbankfin.2017.06.013

[2] Föllmer, H., Schied, A.: Stochastic Finance: An Introduction in Discrete Time, 3rd edn. Walter de Gruyter, Berlin, Germany (2011)

[3] Tsanakas, A.: Dynamic capital allocation with distortion risk measures. Insur. Math. Econ. **35**, 223–243 (2004) https://doi.org/10.1016/j.insmatheco.2003.09.005

[4] Dhaene, J., Tsanakas, A., Valdez, E., Vanduffel, S.: Optimal capital allocation principles. J. Risk Insur. **79**(1), 1–28 (2012) https://doi.org/10.1111/j.1539-6975.2011.01408.x

[5] Landsman, Z., Valdez, E.: Tail conditional expectations for elliptical distributions. N. Am. Actuar. J. **7**, 55–71 (2003) https://doi.org/10.1080/10920277.2003.10596118

[6] Fang, K.T., Kotz, S., Ng, K.W.: Symmetric Multivariate and Related Distributions. Chapman and Hall, London, England (1990)

[7] Li, P., Yin, C.: The tail mean-variance optimal capital allocation under the extended skew-elliptical distribution. J. Comput. Appl. Math. **448**, 115965 (2024) https://doi.org/10.1016/j.cam.2024.115965
